# PRDX5 Regulates Mitochondrial Function and Nuclear Spreading in Myogenesis and Acts With PRDX3 to Delay Muscle Aging

**DOI:** 10.1002/jcsm.70098

**Published:** 2025-10-28

**Authors:** Joonho Suh, Je‐Hyun Eom, Jongmin Baik, Wonn Shim, Max A. Tischfield, Hyun Ae Woo, Yun‐Sil Lee

**Affiliations:** ^1^ Department of Molecular Genetics, School of Dentistry and Dental Research Institute Seoul National University Seoul Republic of Korea; ^2^ Department of Life Science Dongguk University‐Seoul Goyang Republic of Korea; ^3^ Department of Cell Biology and Neuroscience Rutgers University Piscataway New Jersey USA; ^4^ Keck Center for Collaborative Neuroscience Rutgers University Piscataway New Jersey USA; ^5^ Graduate School of Pharmaceutical Sciences, College of Pharmacy Ewha Womans University Seoul Republic of Korea

**Keywords:** myonuclear distribution, mitochondrial dysfunction, muscle aging, PRDX3, PRDX5

## Abstract

**Background:**

Skeletal muscle aging is associated with oxidative stress and mitochondrial dysfunction. Peroxiredoxins (PRDXs), particularly PRDX3 and PRDX5, are antioxidant enzymes that are uniquely localized to mitochondria. While PRDX3 has been reported to play a role in maintaining mitochondrial function in muscle, the specific function of PRDX5 in muscle remains unclear. This study investigated the role of PRDX5 in mitochondrial function, myonuclear distribution and muscle aging.

**Methods:**

Myoblasts were isolated from wild‐type (WT), *Prdx3* ^*−/−*^, *Prdx5* ^*−/−*^ and *Prdx3* ^*−/−*^; *Prdx5* ^*−/−*^ mice crossed with mitochondria reporter (mt‐GFP) mice. Nuclear and mitochondrial positioning were evaluated using confocal and super‐resolution lattice structured illumination microscopy (SIM). Mitochondrial function was assessed by Seahorse oxygen consumption rates (OCR) assays. In vivo analyses included grip strength, treadmill performance and histological evaluation following venom‐induced muscle injury.

**Results:**

During myogenesis, *Prdx5* ^*−/−*^ and *Prdx3* ^*−/−*^; *Prdx5* ^*−/−*^ myotubes exhibited impairments in nuclear spreading, characterized by clustered nuclei, unlike the even distribution observed in WT and *Prdx3* ^*−/−*^ myotubes (44.4% and 44.9% vs. 17.1% and 21.9%, respectively; *p* < 0.001). Mitochondrial ATP production was significantly reduced in *Prdx3* ^*−/−*^, *Prdx5* ^*−/−*^ and *Prdx3* ^*−/−*^; *Prdx5* ^*−/−*^ myotubes (*p* < 0.05). The expression of *Rhot1* and *Trak1*, key regulators of mitochondrial transport, was significantly decreased in *Prdx5* ^*−/−*^ and *Prdx3* ^*−/−*^; *Prdx5* ^*−/−*^ myotubes (*p* < 0.01). Knockdown of *Rhot1* or *Trak1* in WT myotubes led to myonuclear clustering similar to that observed in *Prdx5*‐deficient myotubes, supporting that PRDX5 facilitates mitochondrial transport and nuclear positioning, at least in part, through transcriptional regulation of genes including *Rhot1* and *Trak1*. In vivo, 48‐week‐old *Prdx5* ^*−/−*^ mice exhibited mitochondrial dysfunction and myonuclear clustering in myofibers, with reduced treadmill performance (*p* < 0.05). Muscle regeneration was impaired in *Prdx5* ^*−/−*^ mice, with decreased expression of regeneration and mitochondrial transport markers and increased nuclear clustering in regenerating myofibers (*p* < 0.05). *Prdx3* ^*−/−*^; *Prdx5* ^*−/−*^ double‐knockout mice displayed accelerated muscle aging, including decreased muscle mass and strength, and elevated expression of E3 ligases Atrogin1 and MuRF1 as early as 10 weeks of age (*p* < 0.05). These mice also exhibited increased mitochondrial H_2_O_2_ production, which upregulated the expression of Atrogin1 and MuRF1 (*p* < 0.05).

**Conclusions:**

Our findings reveal a previously unidentified role of PRDX5 in coordinating mitochondrial function and nuclear positioning during myogenesis and muscle regeneration. The combined deficiency of PRDX3 and PRDX5 accelerates muscle aging by exacerbating oxidative stress and mitochondrial dysfunction, suggesting that enhancing their activity may be a promising therapeutic strategy to prevent sarcopenia and age‐related muscle degeneration.

## 1 Introduction

Skeletal muscle is a highly dynamic tissue that relies on efficient mitochondrial function for proper development, regeneration and performance [1]. Mitochondria play a crucial role in energy production and redox homeostasis, both of which are essential for effective muscle contraction and recovery following injury [2, 3, 4]. However, mitochondria are also the primary source of reactive oxygen species (ROS), and excessive ROS production can lead to oxidative stress, which is widely recognized as a major contributor to muscle aging and sarcopenia, a condition characterized by the progressive loss of muscle mass and function in aging individuals [5, 6]. Elevated ROS levels, in turn, induce mitochondrial dysfunction, which has been reported to be associated with various muscle pathologies, including sarcopenia, muscular dystrophies and muscle dysfunction [4, 5, 6, 7, 8]. Therefore, effective antioxidant systems are crucial for neutralizing ROS and preserving mitochondrial health to prevent muscle degeneration during aging.

Mitochondria need to be properly transported and distributed within cells to meet localized energy demands, ensuring efficient ATP delivery and ROS detoxification to minimize oxidative damage [9, 10]. Mammalian mitochondrial Rho 1 (Miro1), an outer mitochondrial membrane protein, and Milton, an adaptor protein that links Miro1 to motor proteins, are key components of the mitochondrial transport process [11]. Specifically, Milton interacts with kinesin motors at one end and binds to Miro1 at the other, forming a complex that effectively loads mitochondria onto the kinesin transport machinery. This Miro1/Milton/kinesin complex facilitates the anterograde transport of mitochondria along the microtubule network for their proper distribution within cells [11]. The critical role of this transport system has been well established in the nervous system, where deficiencies in Miro1 or Milton have been associated with severe neurological diseases and impaired axonal outgrowth [12, 13]. While defective mitochondrial transport has been extensively studied in the context of neurological disorders [14], its significance in myotubes and myofibers, where proper mitochondrial distribution is likely crucial for preventing localized energy deficits and oxidative stress, remains largely unexplored.

During myogenesis, proper positioning of nuclei along the length of myotubes and myofibers is essential to ensure optimal transcriptional regulation and efficient cellular function [15, 16]. Throughout myotube formation, nuclei undergo a series of well‐coordinated movements, including centration and spreading [17]. Nuclear centration occurs immediately after myoblast fusion, during which nuclei migrate towards the center of the newly formed myotube. Following centration, nuclei undergo spreading along the longitudinal axis of the myotube to achieve an even distribution. This step is mediated by kinesin motor proteins, which transport nuclei apart towards the plus ends of microtubules, resulting in the uniform spacing of nuclei along the myotube [16, 17]. This even distribution of nuclei is crucial for adequately supporting the transcriptional and metabolic demands of the surrounding cytoplasm. Indeed, defective nuclear positioning has been linked to a range of muscle pathologies, including muscle dysfunction, muscular dystrophy and centronuclear myopathies [15, 16, 17, 18], indicating that precise nuclear positioning is fundamental for proper myotube formation and muscle function. Since transporting nuclei within muscle cells demands energy, the role of mitochondria is likely crucial. However, the influence of mitochondrial function and transport on nuclear distribution during myotube formation remains largely unknown, requiring further investigation.

Peroxiredoxins (PRDXs) are a family of antioxidant enzymes that play a central role in neutralizing H_2_O_2_ and maintaining cellular redox balance [19]. Among the six isoforms, PRDX3 and PRDX5 are unique in their localization to mitochondria, suggesting that they may have specialized functions in regulating mitochondrial function in muscle tissue. Previous studies have highlighted the role of PRDX3 in maintaining mitochondrial integrity and muscle contractile function [2, 20]. Notably, transgenic mice designed to overexpress *Prdx3* specifically in muscle were shown to reverse mitochondrial dysfunction and prevent muscle wasting induced by oxidative stress [20]. In contrast to the relatively well‐documented functions of PRDX3 in muscle, the specific roles of PRDX5 in muscle remain largely unexplored. Recent evidence suggests that PRDX5 extends its functions beyond antioxidant activity by regulating gene expression through direct interactions with transcription factors in both the cytoplasm and nucleus [21, 22], raising the possibility that PRDX5 may regulate mitochondrial function and nuclear distribution in muscle cells through transcriptional mechanisms.

In this study, we investigated the role of PRDX5 in skeletal muscle by utilizing *Prdx5* ^*−/−*^ and *Prdx3* ^*−/−*^ *; Prdx5* ^*−/−*^ mouse models. We found that PRDX5 is essential for maintaining mitochondrial energy production, transport and proper myonuclear distribution during myogenesis and muscle regeneration. Mechanistically, PRDX5 promotes the expression of mitochondrial transport regulators Miro1 and Milton, which are crucial for effective mitochondrial transport and myonuclear spreading. Furthermore, we demonstrate that the combined deficiency of PRDX3 and PRDX5 leads to accelerated muscle aging, characterized by enhanced oxidative stress, mitochondrial dysfunction and muscle atrophy. Our findings reveal a previously unrecognized role of PRDX5 in regulating mitochondrial function and nuclear distribution in skeletal muscle and suggest that enhancing PRDX3 and PRDX5 function may represent a promising therapeutic strategy for preventing age‐related muscle degeneration.

## 2 Materials and Methods

### 2.1 Mice

All mice were maintained on a C57BL/6 J background, and all animal studies were approved by the Institutional Animal Care and Use Committees at Seoul National University. *Prdx3* ^*−/−*^ and *Prdx5* ^*−/−*^ mice have been previously described [23, 24]. *Prdx3* ^*−/−*^ and *Prdx5* ^*−/−*^ mice were crossed to generate *Prdx3* ^*−/−*^; *Prdx5* ^*−/−*^ mice. Conditional mitochondria reporter mice, which express GFP in mitochondria upon crossing with Cre mice [25], were kindly provided by Jeremy Nathans (Johns Hopkins University School of Medicine, MD). *EIIA‐Cre* mice, which ubiquitously express Cre recombinase, were crossed with the conditional mitochondria reporter mice to obtain mt‐GFP mice. Subsequently, *Prdx5* ^*−/−*^ and *Prdx3* ^*−/−*^; *Prdx5* ^*−/−*^ mice were crossed with mt‐GFP mice to generate *Prdx5* ^*−/−*^; mt‐GFP and *Prdx3* ^*−/−*^; *Prdx5* ^*−/−*^; mt‐GFP mice, respectively. Genotyping primers are listed in Table S1.

### 2.2 Muscle Function

Maximal forelimb grip strength was measured using a Grip Strength Meter (Bioseb) according to the manufacturer's guidelines. Mice were allowed to grasp a metal bar using their forelimbs only and were pulled backward horizontally. The test was performed in five consecutive trials per session, and the highest recorded value was noted. Each mouse underwent three sessions, and the average of the three sessions was used for analysis. The running endurance test was conducted using a treadmill (JD‐A‐09, Jeungdo Bio & Plant), following a previously published protocol [26]. Briefly, the treadmill speed was increased by 2–3 m/min every 3 min, and the incline was raised by 5% at 6, 12 and 21 min after the start. Work was calculated as the product of force (mass × gravitational acceleration × sin [angle]) and speed.

### 2.3 Muscle Regeneration

Snake venom from *Sepedon hemachatus* (Sigma) was prepared as a 10‐μM solution and injected into the right gastrocnemius (GAS) and tibialis anterior (TA) muscles of 4‐week‐old mice under isoflurane anaesthesia. A total of 80 μL was administered into the GAS and 30 μL into the TA. Cross‐sections of the GAS muscle were prepared using a CM1860 cryostat (Leica Biosystems) and stained with either haematoxylin and eosin (H&E) or immunofluorescence. Immunofluorescence staining was performed using an anti–embryonic myosin heavy chain antibody (BF‐G6, DSHB; 1:20) and an anti–laminin antibody (L9393, Sigma; 1:500). H&E and immunofluorescence staining were conducted following the previous protocol [27]. Muscle regeneration was also assessed by analysing gene expression in regenerating TA muscles.

### 2.4 Dual‐Energy X‐Ray Absorptiometry

Dual‐energy x‐ray absorptiometry (DXA) scanning was performed using the InAlyzer scanner (Medikors), and analysis was conducted with InAlyzer software (Medikors) following the previously described method by our group [28].

### 2.5 In Vitro Myogenesis

Primary myoblasts were isolated using the preplating method. Briefly, muscle tissues (quadriceps, GAS, and TA) from adult mice were minced and digested in a mixture of Collagenase type IV (Gibco) and Dispase II (Gibco) for approximately 1 h on a rocker at 37°C. The cell suspension was then preplated on a plastic dish for 1 h, allowing fibroblasts to adhere while myoblasts remained in suspension. The supernatant containing myoblasts was transferred to a new dish for another 1‐h preplating step, which was repeated 5 times in total. After the final step, myoblasts were plated on a 0.1% gelatin‐coated dish. Myoblasts were expanded in F‐10 medium (Gibco) supplemented with 20% horse serum (Gibco), penicillin–streptomycin (Gibco) and 2.5 ng/mL fibroblast growth factor (FGF) basic (PeproTech). Differentiation into myotubes was induced by plating myoblasts onto glass‐bottom dishes or plates coated with 1 mg/mL Matrigel (Corning) in DMEM (Cytiva) containing 5% horse serum and penicillin–streptomycin. At 24 h of differentiation, myotubes were treated with either 0.5 mM H_2_O_2_ (Daejung) or 0.1 μM Oligomycin A (Sigma) to analyse nuclear clustering. Fixed myotubes were stained with Phalloidin probes (Invitrogen) to label actin and DAPI to visualize nuclei.

### 2.6 Transfection

For live imaging, myoblasts were transfected with a *Map 7* or Lifeact plasmid tagged with mCherry using FuGENE HD (Promega) or Lipofectamine 3000 (Invitrogen) transfection reagent to label microtubules or actin filaments, respectively. The pCMV‐*Map 7* plasmid was obtained from the Korea Human Gene Bank, Medical Genomics Research center, KRIBB, Korea. The pCMV‐mCherry‐Lifeact plasmid was kindly provided by Jin Man Kim (Seoul National University, Korea). pCMV‐*Rhot1* and pCMV‐*Trak1* plasmids (Origene; MR209606 and MR209421, respectively) were transfected using Lipofectamine 3000 (Invitrogen) reagent. For siRNA transfection, Lipofectamine RNAiMAX (Invitrogen) transfection reagent was used. Predesigned siRNAs (Bioneer) were used for siCtrl (SN‐1002), si*Rhot1* (59 040‐1) and si*Prdx5* (54 683‐1). The sequences for si*Trak1* (Bioneer) are as follows: Sense: GAUGACACAGGUGACCACA; Antisense: UGUGGUCACCUGUGUCAUC. All transfections were performed according to the manufacturer's protocol.

### 2.7 Single Myofiber Analysis

Single myofibers were isolated from extensor digitorum longus (EDL) muscles by digesting the tissue in 2 mg/mL Collagenase type 1 (Sigma) solution for approximately 1 h at 37°C. Digested tissues were then gently triturated using a wide‐bore pipette to release individual myofibers. Fibre immunostaining was performed following a previously published protocol with slight modifications [29]. Anti‐PAX7 antibody (DSHB; 1:50) and conjugated alpha‐bungarotoxin (Invitrogen; 1:100) were used to label satellite cells and neuromuscular junctions, respectively.

### 2.8 Fluorescence Imaging

Myoblasts, myotubes and muscle cross‐sections were imaged using the Zeiss LSM 800 confocal microscope (Zeiss) with or without Airyscan mode. Live imaging of myoblast differentiation was performed using Zeiss Axio Observer Z1 (Zeiss), Zeiss LSM 800 confocal microscope (Zeiss), Zeiss Elyra 7 with Lattice SIM (Zeiss), or Zeiss Lattice SIM 5 (Zeiss) and processed using the Zeiss Zen blue edition software (Zeiss). Tetramethylrhodamine (TMRM) and mitochondrial GFP intensities within defined regions of interest (Figure 4) were quantified using the Zeiss Zen blue edition software (Zeiss). TMRM signal intensity was normalized to the corresponding mitochondrial GFP signal. Mitochondrial GFP intensity profiles were generated using the Fiji software.

### 2.9 Mitochondrial Function Analysis

Mitochondrial membrane potential in myotubes or muscle cross‐sections was assessed after staining with 100 nM TMRM (Invitrogen) for 30 min at 37°C, following the manufacturer's protocol. Mitochondrial oxygen consumption rate (OCR) was measured using the Seahorse XFe96 Analyzer (Agilent) following the manufacturer's protocol and our previously described method [30]. After basal readings were recorded, 1.5 μM oligomycin, 0.5 μM FCCP and 0.5 μM rotenone/antimycin A were sequentially injected. All reagents were provided in the Cell Mito Stress Test Kit for XFe/XF Analyzers (Agilent). Seahorse data were normalized to protein content, which was measured using the Bradford assay. H_2_O_2_ production by isolated mitochondria was assessed using the Amplex Red Hydrogen Peroxide/Peroxidase Assay Kit (Invitrogen), following the manufacturer's instructions. Briefly, GAS tissues were minced and treated with 0.3 mg/mL Nagarse (Sigma) for 3 min on ice, followed by centrifugation at 700 × g for 10 min, repeated twice to remove debris. Mitochondria were pelleted by centrifugation at 8000 × g for 10 min. Isolated mitochondria (30 μg) were incubated with Amplex Red reagent and HRP solution at room temperature, and fluorescence was measured using a Spark 10 M microplate reader (Tecan).

### 2.10 Quantitative Reverse Transcriptase Polymerase Chain Reaction

Total RNA was extracted using the QIAzol Lysis Reagent (Qiagen) and the AccuPrep Universal RNA Extraction Kit (Bioneer). cDNA was synthesized using the PrimeScript RT Reagent Kit (Takara) according to the manufacturer's guidelines. Quantitative reverse transcriptase polymerase chain reaction (qRT‐PCR) was performed using the TB Green Premix Ex Taq II (Takara) on a QuantStudio 3 Real‐Time PCR system (Applied Biosystems). Relative gene expression was determined using the 2^(−∆∆Ct)^ method, normalized to 18S rRNA. For the evaluation of mitochondrial DNA (mtDNA) content, qRT‐PCR was performed on purified DNA using primers for *Cytb* and *Rplp0* (36B4). A complete list of primers used in this study is provided in Table S2.

### 2.11 Statistical Analysis

All values were presented as mean ± standard error of the mean (SEM). Statistical analysis for comparisons involving more than 2 groups was performed using one‐way ANOVA followed by Tukey's post hoc test, while comparisons between 2 groups were analysed using Student's *t* test. A *p* value of less than 0.05 was considered statistically significant. All statistical analyses were conducted using the SPSS Statistics 26 (IBM) software.

## 3 Results

### 3.1 *Prdx3* and *Prdx5* Exhibit Distinct Expression Patterns Compared to Other *Prdx* Genes During Myogenesis and Muscle Regeneration

**FIGURE 1:**
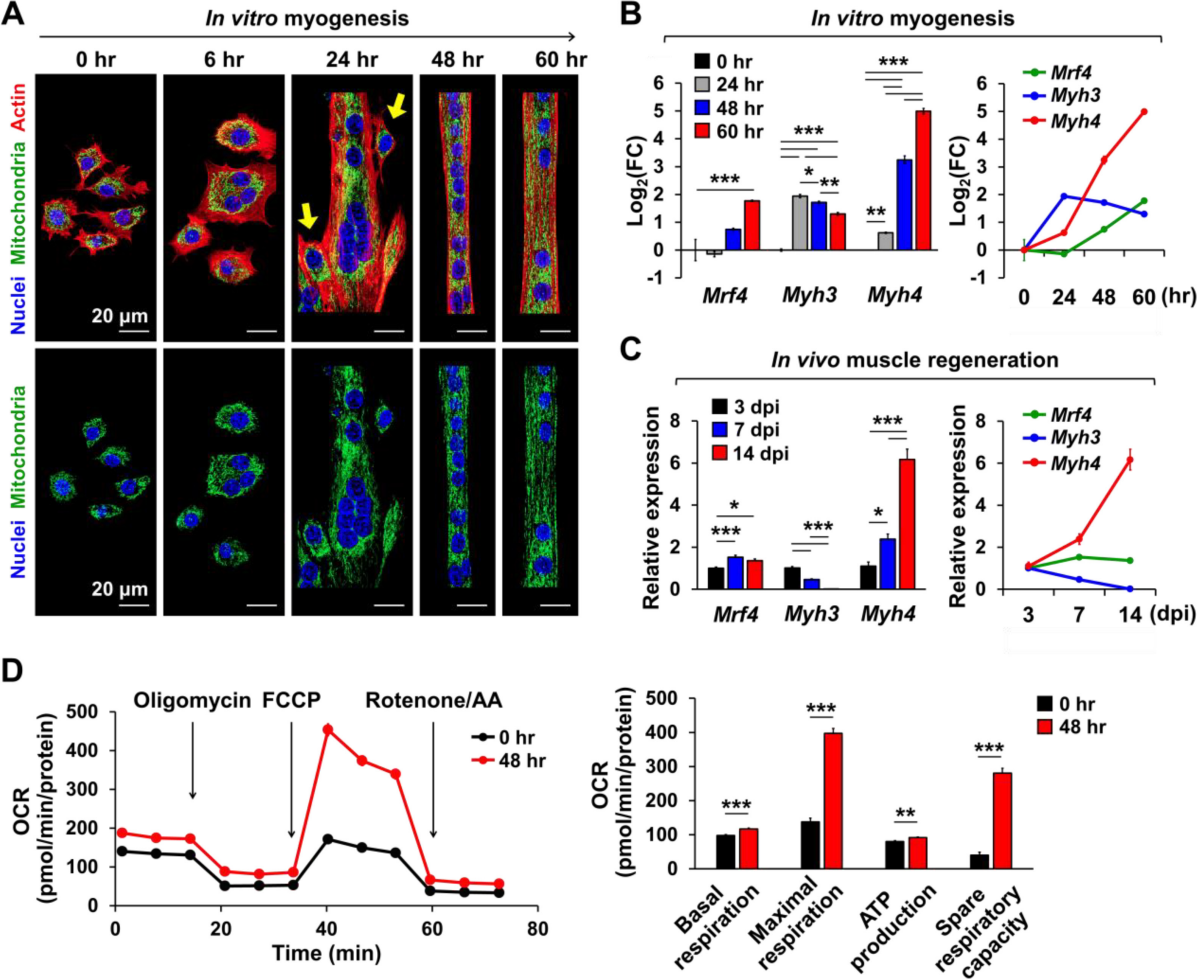
Comparison of myogenic marker expression patterns during in vitro myogenesis and in vivo muscle regeneration. (A) Representative confocal images illustrating the differentiation of primary myoblasts into myotubes. Arrows on 24 h of differentiation (24 h) highlight fusing myoblasts. From 24 to 60 h, nuclei and mitochondria are longitudinally distributed along the myotubes. Scale bars are displayed with actual size values. (B, C) qRT‐PCR analysis of myogenic markers during in vitro myogenesis (B, *n* = 3) and in vivo muscle regeneration induced by snake venom (C, *n* = 5–7). Note the similar expression patterns of myogenic markers between in vitro myogenesis and in vivo muscle regeneration. Dpi, days after injury. (D) OCR analysis during in vitro myogenesis (*n* = 8). Note that mitochondrial function parameters such as basal and maximal respiration, ATP production and spare respiratory capacity are elevated in mature myotubes. All data represent mean ± SEM. Statistical significance is indicated as *p < 0.05, **p < 0.01 and ***p < 0.001, analysed by ANOVA with Tukey's post hoc test (B, C), or by *t* test (D). OCR, oxygen consumption rate; qRT‐PCR, quantitative reverse transcriptase polymerase chain reaction.

To analyse nuclear and mitochondrial positioning during myotube differentiation, we isolated primary myoblasts from mitochondrial reporter (mt‐GFP) mice, which express green fluorescent protein (GFP) in the mitochondrial matrix, and induced differentiation into myotubes. Myoblast fusion was observed as early as 6 h into differentiation, with notable nuclear centration occurring by 24 h (Figure 1A). By 48 h, elongated myotubes with evenly distributed nuclei and mitochondria were observed (Figure 1A and Movie 1). At 24 h, when nuclei and mitochondria began to actively disperse along the myotube, the expression of *Mrf4* and *Myh4*, mid‐ to late‐stage myogenic markers, sharply increased, whereas the expression of *Myh3*, an early myogenic marker, decreased (Figure 1B). A similar expression pattern of myogenic markers was detected in regenerating muscle in vivo following snake venom‐induced injury to the TA muscle (Figure 1C). Mitochondrial OCR significantly increased during myogenesis, as indicated by elevated basal and maximal respiration, ATP production, and spare respiratory capacity at 48 h compared to 0 h of myogenic induction (Figure 1D).

**FIGURE 2:**
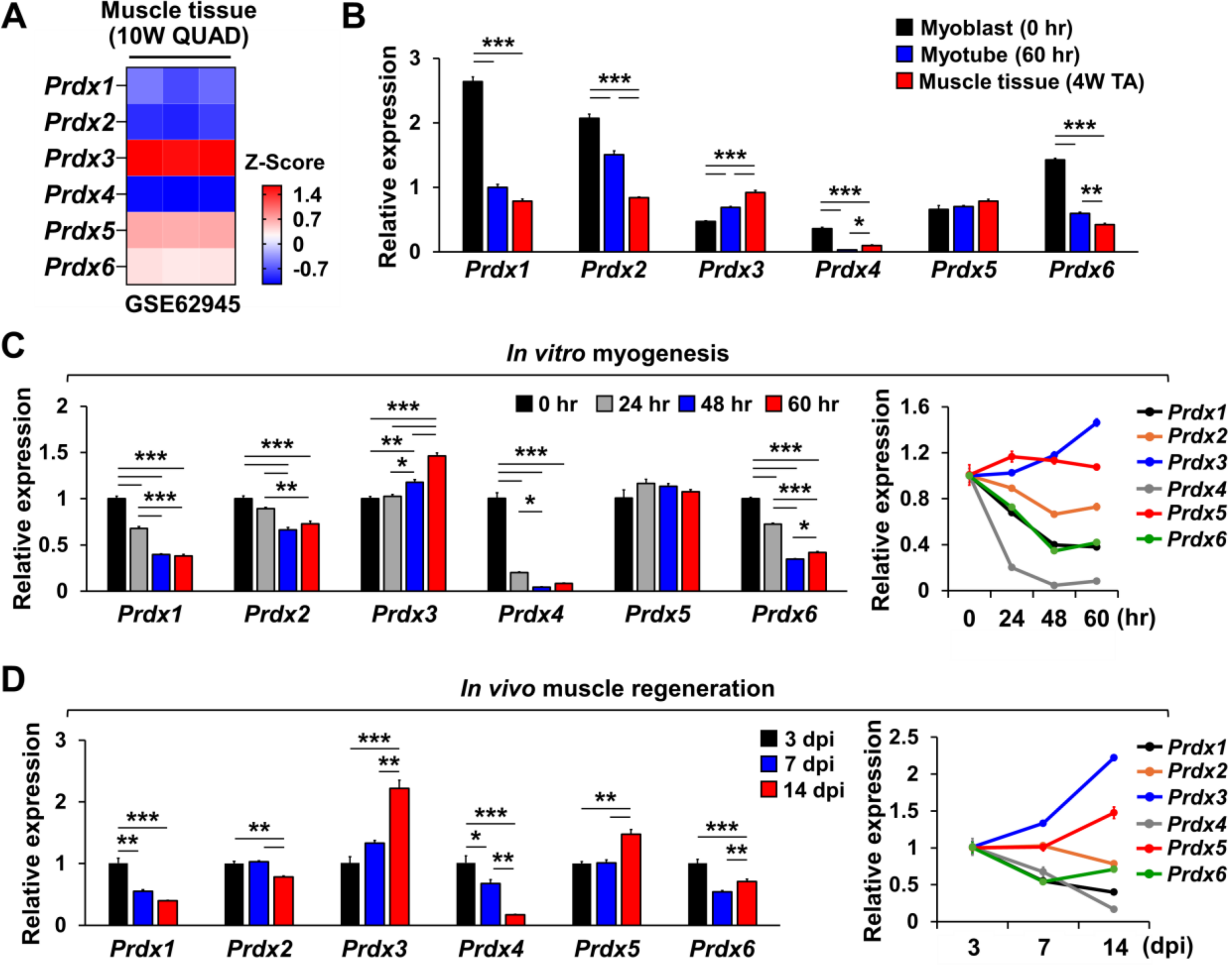
*Prdx3* and *Prdx5* exhibit distinct expression patterns in developing myotubes and regenerating skeletal muscle compared to other *Prdx* genes. (A) Comparison of *Prdx* gene expression levels in quadriceps muscles of 10‐week‐old mice (GSE62945). Note that *Prdx3* and *Prdx5* are among the most highly expressed *Prdx* genes in muscle tissue. (B) qRT‐PCR analysis of *Prdx* expression levels in cultured myoblasts, cultured myotubes and tibialis anterior (TA) muscle tissues of 4‐week‐old mice (*n* = 3). Expression levels are shown relative to the *Prdx1* level in myotubes. Among the *Prdx* genes, only *Prdx3* and *Prdx5* exhibit greater or comparable expression levels in myotubes and muscle tissues compared to myoblasts. (C, D) qRT‐PCR analysis of *Prdx* expression patterns during in vitro myogenesis (C, *n* = 3) and in vivo muscle regeneration induced by snake venom (D, *n* = 3). Notably, *Prdx3* and *Prdx5* show an increasing expression pattern during muscle regeneration, whereas other *Prdx* genes exhibit a decreasing expression pattern. All data represent mean ± SEM. Statistical significance is indicated as *p < 0.05, **p < 0.01 and ***p < 0.001, analysed by ANOVA with Tukey's post hoc test. *Prdx*, peroxiredoxin; qRT‐PCR, quantitative reverse transcriptase polymerase chain reaction; QUAD, quadriceps.

Next, we examined the expression patterns of *Prdx* genes in skeletal muscle tissue and in vitro myotubes. In the quadriceps muscle of 10‐week‐old mice, *Prdx3* and *Prdx5* were the two most abundantly expressed *Prdx* isoforms (Figure 2A). Furthermore, a comparison of *Prdx* gene expression levels in myoblasts, in vitro myotubes and TA muscle revealed that only *Prdx3* and *Prdx5* showed an increasing (*Prdx3*) or a stable (*Prdx5*) expression pattern as myoblasts differentiated into myotubes and matured into muscle tissue, whereas the expression of other *Prdx* genes significantly decreased (Figure 2B). A similar trend was observed during in vitro myogenesis, where only *Prdx3* and *Prdx5* displayed increasing or stable expression levels (Figure 2C). Among all *Prdx* genes, *Prdx3* and *Prdx5* were also the only ones to show increased expression during in vivo muscle regeneration induced by snake venom (Figure 2D). These findings suggest that *Prdx3* and *Prdx5* may have distinct and functionally significant roles in myogenesis and muscle regeneration.

### 3.2 PRDX5 Deficiency Impairs Myonuclear Distribution

**FIGURE 3:**
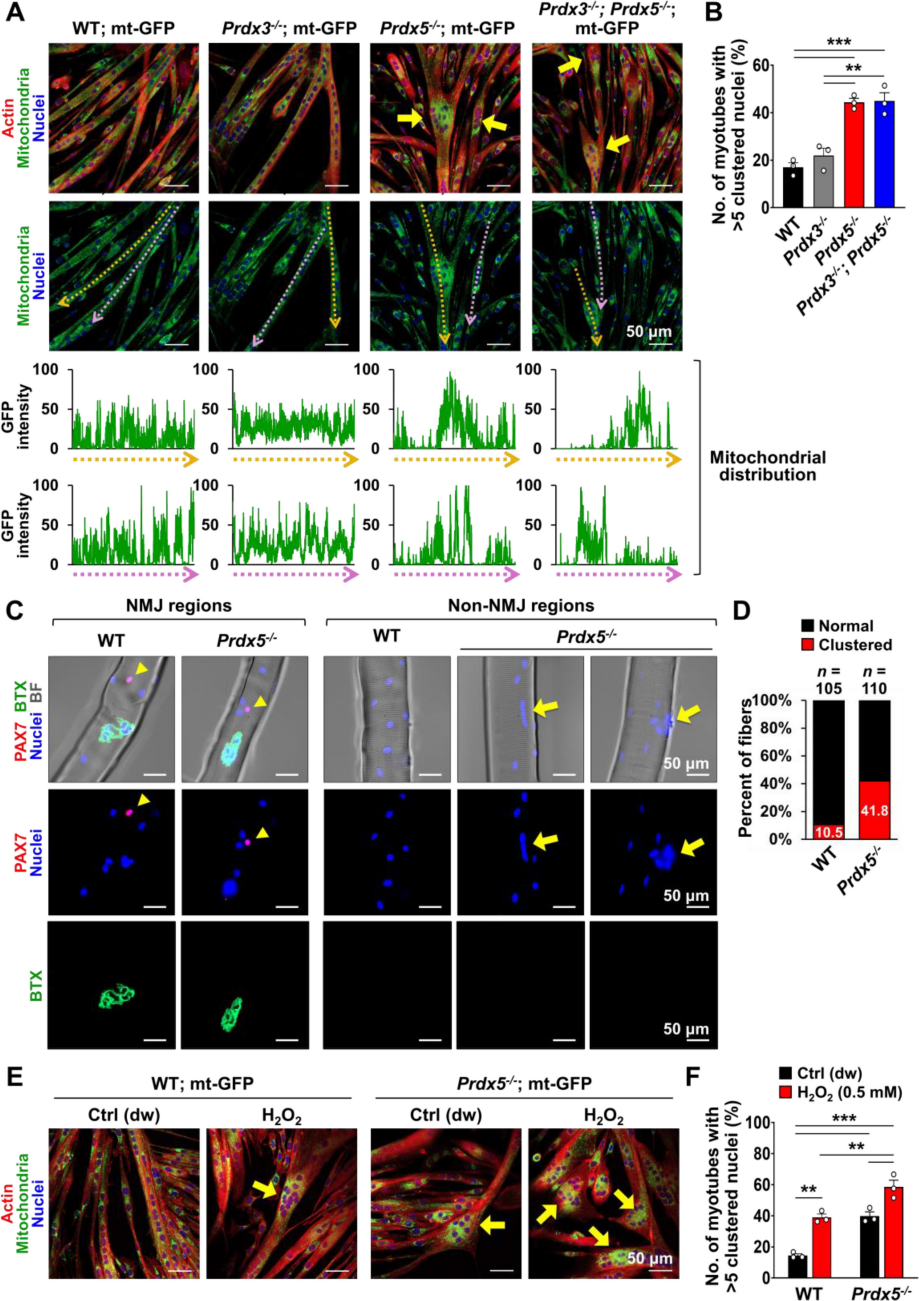
PRDX5 deficiency impairs nuclear and mitochondrial distribution during myogenesis. (A) Representative confocal images of wild‐type mitochondrial reporter mice expressing GFP in the mitochondrial matrix (WT; mt‐GFP), *Prdx3* ^*−/−*^; mt‐GFP, *Prdx5* ^*−/−*^; mt‐GFP and *Prdx3* ^*−/−*^; *Prdx5* ^*−/−*^; mt‐GFP myotubes at 48 h. Arrows indicate abnormal myotubes with clustered nuclei or mitochondria. Mitochondrial GFP intensity profiles along the yellow and purple dashed lines highlight mitochondrial distribution patterns within individual myotubes. Note the uneven mitochondrial distribution in *Prdx5* ^*−/−*^; mt‐GFP and *Prdx3* ^*−/−*^; *Prdx5* ^*−/−*^; mt‐GFP myotubes. (B) Quantitative analysis of clustered nuclei in myotubes at 48 h. The percentage of myotubes containing more than 5 clustered nuclei was measured (*n* = 3 independent wells; 106–114 myotubes). The absence of *Prdx3* did not further increase myonuclear clustering in *Prdx5* ^*−/−*^ myotubes. (C, D) Representative fluorescence images (C) and quantitative analysis (D) of WT and *Prdx5* ^*−/−*^ myofibers isolated from EDL muscles of 48‐week‐old mice. In (C), arrowheads and arrows indicate PAX7‐positive satellite cells and clustered myonuclei outside of NMJ regions, respectively. Myofibers were classified as having clustered myonuclei in (D) if they contained at least three adjacently located myonuclei. A total of 105 and 110 myofibers were analysed for WT and *Prdx5* ^*−/−*^ groups, respectively, with myofibers pooled from three mice per group. BTX, bungarotoxin. (E, F) Representative confocal images (E) and quantitative analysis (F) of WT; mt‐GFP and *Prdx5* ^*−/−*^; mt‐GFP myotubes treated with distilled water (dw) or 0.5 mM H_2_O_2_, 24 h after myogenic induction (*n* = 3 independent wells; 94–102 myotubes). In (E), arrows highlight clustered nuclei. The percentage of myotubes containing more than five clustered nuclei was measured in (F). All scale bars are displayed with actual size values. Data (B) and (F) represent mean ± SEM. Statistical significance is indicated as **p < 0.01 and ***p < 0.001, analysed by ANOVA with Tukey's post hoc test. EDL, extensor digitorum longus; NMJ, neuromuscular junction.

To investigate the role of PRDX3 and PRDX5 in myogenesis, we isolated myoblasts from wild‐type mt‐GFP (WT; mt‐GFP), *Prdx3* ^*−/−*^; mt‐GFP, *Prdx5* ^*−/−*^; mt‐GFP and *Prdx3* ^*−/−*^; *Prdx5* ^*−/−*^; mt‐GFP mice and induced differentiation into myotubes. After 48 h of differentiation, *Prdx5* ^*−/−*^; mt‐GFP and *Prdx3* ^*−/−*^; *Prdx5* ^*−/−*^; mt‐GFP myotubes exhibited impaired nuclear and mitochondrial distribution, leading to aggregated nuclei and mitochondria, while WT; mt‐GFP and *Prdx3* ^*−/−*^; mt‐GFP myotubes displayed an even distribution of nuclei and mitochondria (Figure 3A, Movies 2, 3). Quantification of myotubes containing clustered nuclei revealed that *Prdx5* ^*−/−*^ myotubes exhibited more than twice the percentage of clustered nuclei compared to WT myotubes (Figure 3B). Notably, nuclear clustering was not further increased in *Prdx3* ^*−/−*^ or *Prdx3* ^*−/−*^; *Prdx5* ^*−/−*^ myotubes compared to WT and *Prdx5* ^*−/−*^ myotubes, respectively, indicating that the impaired nuclear distribution is specifically caused by *Prdx5* deficiency rather than *Prdx3* deficiency (Figure 3B). Next, we investigated whether myonuclear distribution is impaired in mature myofibers of *Prdx5* ^*−/−*^ mice in vivo (Figure 3C). Myofibers were isolated from EDL muscles of 48‐week‐old mice and stained for neuromuscular junctions (NMJs) and satellite cells. Since myonuclei are naturally clustered in NMJs [31], we avoided these regions for analysis. Additionally, we separately labelled satellite cells using a PAX7 antibody to effectively distinguish them from myonuclei. Analysis of myofibers revealed a dramatic, 4‐fold increase in the percentage of myofibers with clustered myonuclei in *Prdx5* ^*−/−*^ myofibers (Figure 3D), indicating that *Prdx5* deficiency disrupts myonuclear distribution both during in vitro myogenesis and in mature myofibers in vivo.

Because PRDXs are well‐known H_2_O_2_ scavengers and have been reported to prevent ROS accumulation [2, 32], we treated developing myotubes with H_2_O_2_ to assess its effect on nuclear clustering. Treatment with 0.5 mM H_2_O_2_ at 24 h of myogenesis significantly increased nuclear clustering in WT myotubes at 48 h, reaching a level comparable to that observed in *Prdx5* ^*−/−*^ myotubes (Figure 3E, F). However, H_2_O_2_ treatment further exacerbated nuclear clustering in *Prdx5* ^*−/−*^ myotubes (Figure 3E, F), suggesting that although H_2_O_2_ accumulation may contribute to the myonuclear clustering phenotype in *Prdx5* ^*−/−*^ myotubes, it is not the sole cause.

### 3.3 PRDX5 Deficiency Leads to Mitochondrial Dysfunction in Skeletal Muscle

**FIGURE 4:**
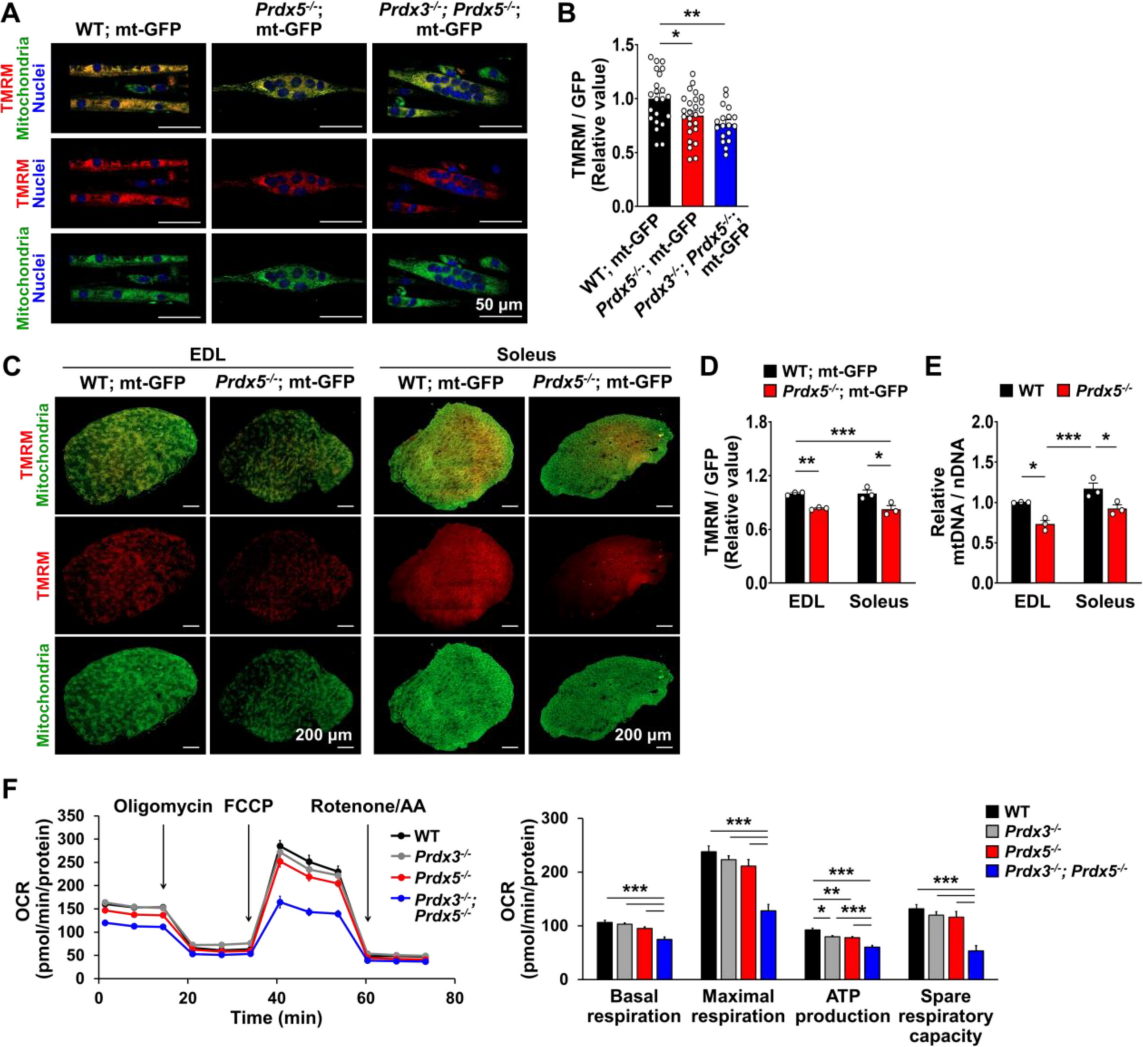
Absence of PRDX5 leads to mitochondrial dysfunction in skeletal muscle. (A, B) Representative confocal images (A) and quantitative analysis (B) of WT; mt‐GFP, *Prdx5* ^*−/−*^; mt‐GFP and *Prdx3* ^*−/−*^; *Prdx5* ^*−/−*^; mt‐GFP myotubes stained with TMRM (tetramethylrhodamine methyl ester, a mitochondrial membrane potential indicator) at 48 h (*n* = 18–25 myotubes from 3 independent wells). (C, D) Representative confocal images (C) and quantitative analysis (D) of TMRM‐stained muscle cross‐sections of 48‐week‐old WT; mt‐GFP and *Prdx5* ^*−/−*^; mt‐GFP mice (*n* = 3). Note the diminished mitochondrial membrane potential in both the EDL and soleus muscles of *Prdx5* ^*−/−*^ mice.( E) qRT‐PCR analysis of the relative ratio of mtDNA to nDNA in EDL and soleus muscle tissues of 48‐week‐old mice (*n* = 3).(F) OCR analysis in myotubes at 72 h (*n* = 9). Note that mitochondrial ATP production is significantly decreased in *Prdx3* ^*−/−*^, *Prdx5* ^*−/−*^ and *Prdx3* ^*−/−*^; *Prdx5* ^*−/−*^ myotubes compared to WT myotubes. All scale bars are displayed with actual size values. All data represent mean ± SEM Statistical significance is indicated as *p < 0.05, **p < 0.01 and ***p < 0.001, analysed by ANOVA with Tukey's post hoc test. mtDNA, mitochondrial DNA; nDNA, nuclear DNA.

We next examined whether *Prdx5* deficiency affects mitochondrial function in myotubes and muscle tissue. To assess mitochondrial function, we used TMRM dye to measure mitochondrial membrane potential. At 48 h of differentiation, mitochondrial membrane potential, indicated by TMRM fluorescence intensity normalized to endogenous mitochondrial GFP intensity, was significantly reduced in *Prdx5* ^*−/−*^; mt‐GFP and *Prdx3* ^*−/−*^; *Prdx5* ^*−/−*^; mt‐GFP myotubes compared to WT; mt‐GFP myotubes (Figure 4A, B). Furthermore, mitochondrial membrane potential was significantly lower in both EDL and soleus muscle cross‐sections of 48‐week‐old *Prdx5* ^*−/−*^; mt‐GFP mice compared to WT; mt‐GFP mice (Figure 4C, D). Notably, endogenous mitochondrial GFP signal appeared reduced in muscle cross‐sections of *Prdx5* ^*−/−*^; mt‐GFP mice, implying a decrease in mitochondrial content (Figure 4D). Consistently, quantification of the mitochondrial DNA‐to‐nuclear DNA ratio revealed a reduction in mitochondrial quantity in both the EDL and soleus muscles of *Prdx5* ^*−/−*^ mice compared to WT mice (Figure 4E). We also measured mitochondrial OCR in differentiated myotubes, which revealed a significant reduction in ATP production in *Prdx3* ^*−/−*^ and *Prdx5* ^*−/−*^ myotubes (Figure 4F). Basal respiration, maximal respiration, ATP production and spare respiratory capacity were further reduced in *Prdx3* ^*−/−*^; *Prdx5* ^*−/−*^ myotubes, indicating an additive effect of dual deficiency on mitochondrial function (Figure 4F). Additionally, staining myotubes with ATP‐Red dye, which specifically labels mitochondrial ATP, confirmed that mitochondrial ATP production was diminished in *Prdx5*‐deficient myotubes (Movie 4). Mitochondrial morphology was also abnormal in *Prdx5* ^*−/−*^ and *Prdx3* ^*−/−*^; *Prdx5* ^*−/−*^ myotubes, exhibiting a fragmented appearance (Movie 4). The expression of mitochondrial fusion markers, particularly *Mfn2*, showed a decreasing trend in *Prdx5*‐deficient myotubes, while mitochondrial fission markers remained unchanged (Figure S1).

To investigate whether mitochondrial dysfunction induces nuclear clustering in myotubes, we treated developing myotubes with Oligomycin A, an ATP synthase inhibitor. Treatment with 0.1 μM oligomycin A at 24 h of differentiation dramatically increased nuclear clustering by 48 h (Figure S2), suggesting that mitochondrial ATP production is essential for proper nuclear distribution and that mitochondrial dysfunction partially contributes to impaired nuclear spreading in *Prdx5*‐deficient myotubes.

### 3.4 PRDX5 Induces Miro1 and Milton Expression to Promote Mitochondrial Transport During Myogenesis

**FIGURE 5:**
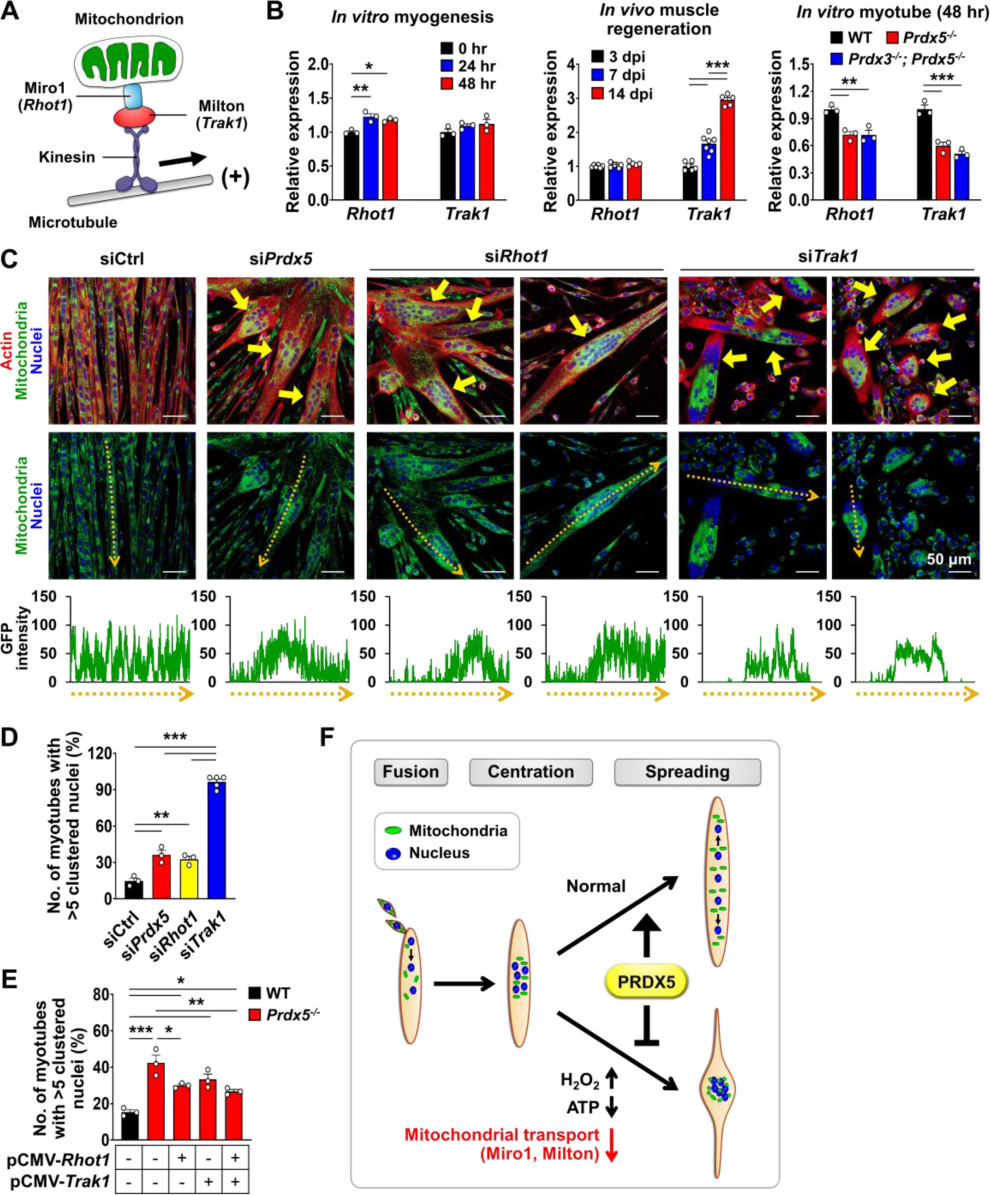
PRDX5 regulates Miro1 and Milton expression to promote mitochondrial transport, facilitating nuclear distribution during myotube formation. (A) Diagram illustrating key proteins involved in mitochondrial transport. Miro1, a mitochondrial outer membrane protein encoded by *Rhot1* gene, interacts with Milton, an adaptor protein encoded by *Trak1* gene, to anchor mitochondria to the motor protein kinesin. (B) qRT‐PCR analysis of mitochondrial transport regulators (*Rhot1* and *Trak1*) during in vitro myogenesis (*n* = 3) and venom‐induced in vivo muscle regeneration (*n* = 5–7), as well as in WT, *Prdx5* ^*−/−*^ and *Prdx3* ^*−/−*^; *Prdx5* ^*−/−*^ myotubes cultured for 48 h (*n* = 3). (C) Representative confocal images of siRNA‐treated myotubes at 48 h. Arrows indicate abnormal myotubes with clustered nuclei or mitochondria. Mitochondrial GFP intensity profiles along the yellow dashed lines highlight mitochondrial distribution patterns within individual myotubes. Scale bars are displayed with actual size values.(D) Quantitative analysis of clustered nuclei in siRNA‐treated myotubes at 48 h (*n* = 3–5 independent wells; 95–110 myotubes). The percentage of myotubes containing more than 5 clustered nuclei was measured. (E) Quantitative analysis of clustered nuclei in *Prdx5* ^*−/−*^ myotubes at 48 h following overexpression of *Rhot1*, *Trak1*, or both (*n* = 3 independent wells; 92–99 myotubes). The percentage of myotubes containing more than 5 clustered nuclei was measured. The pCMV empty vector was used as a control. (F) Summary diagram illustrating how PRDX5 regulates nuclear spreading during myotube formation. PRDX5 induces Miro1 and Milton expression to promote mitochondrial transport, which is essential for proper nuclear distribution. All data represent mean ± SEM Statistical significance is indicated as *p < 0.05, **p < 0.01 and ***p < 0.001, analysed by ANOVA with Tukey's post hoc test.

Inside the cell, mitochondria are actively transported along microtubules by kinesin and dynein motor proteins [11]. In particular, kinesin motor proteins mediate the anterograde transport of mitochondria towards microtubule plus ends, a key process in mitochondrial distribution along myotubes. Mitochondria are linked to kinesin motor proteins via Miro1, a mitochondrial outer membrane protein, and Milton, an adaptor protein that connects kinesin to Miro1 (Figure 5A). During in vitro myotube differentiation, the expression of *Rhot1*, which encodes Miro1, significantly increased at 24 and 48 h compared to 0 h (Figure 5B). Similarly, the expression of *Trak1*, which encodes Milton, exhibited an increasing trend during in vitro myotube formation and was significantly elevated in regenerating muscle tissue in vivo at 7 and 14 days after injury (dpi) (Figure 5B). Notably, the expression of both *Rhot1* and *Trak1* was significantly downregulated in *Prdx5* ^*−/−*^ and *Prdx3* ^*−/−*^; *Prdx5* ^*−/−*^ myotubes at 48 h (Figure 5B). However, *Rhot1* and *Trak1* expression levels were not further reduced in *Prdx3* ^*−/−*^; *Prdx5* ^*−/−*^ myotubes compared to *Prdx5* ^*−/−*^ myotubes, suggesting that PRDX5 specifically regulates their expression levels. Furthermore, treatment with H_2_O_2_ did not decrease *Rhot1* or *Trak1* expression in myotubes, indicating that their downregulation in *Prdx5*‐deficient myotubes is unlikely to be an indirect effect of elevated H_2_O_2_ levels (Figure S3A). The expression of *Kif5b*, which encodes a kinesin subunit, was mildly downregulated in *Prdx5* ^*−/−*^ myotubes (Figure S3B).

To determine whether mitochondrial transport plays a significant role in nuclear distribution during myotube formation, we performed siRNA‐mediated knockdown of *Prdx5*, *Rhot1* or *Trak1* in WT; mt‐GFP myoblasts simultaneously with myogenic induction (Figure 5C). After confirming successful gene knockdown (Figure S3C) and impaired mitochondrial distribution and transport along microtubules (Figure 5C and Movie 5), we assessed nuclear distribution in myotubes at 48 h, which revealed a significant increase in nuclear clustering upon *Prdx5*, *Rhot1* or *Trak1* downregulation (Figure 5D). *Trak1* knockdown led to the most dramatic increase in myonuclear clustering, with approximately 90% of myotubes exhibiting clustered nuclei, while *Rhot1* knockdown resulted in a similar level of nuclear clustering as *Prdx5* knockdown (Figure 5D). *Prdx5*, *Rhot1* or *Trak1* knockdown also significantly reduced the expression of *Mrf4*, a myogenic maturation marker, with the most pronounced reduction observed upon *Trak1* knockdown (Figure S3D). Conversely, overexpression of *Rhot1* alone or in combination with *Trak1* significantly attenuated nuclear clustering in *Prdx5* ^*−/−*^ myotubes, indicating that restoring mitochondrial transport promotes proper nuclear distribution (Figure 5E and Figure S3E). Collectively, these findings suggest that mitochondrial transport via Miro1 and Milton is essential for normal myotube development and nuclear spreading and that PRDX5 facilitates this process by promoting the expression of *Rhot1* and *Trak1* (Figure 5F).

### 3.5 Absence of PRDX5 Leads to Impaired Muscle Regeneration and Mitochondrial Transport In Vivo

**FIGURE 6:**
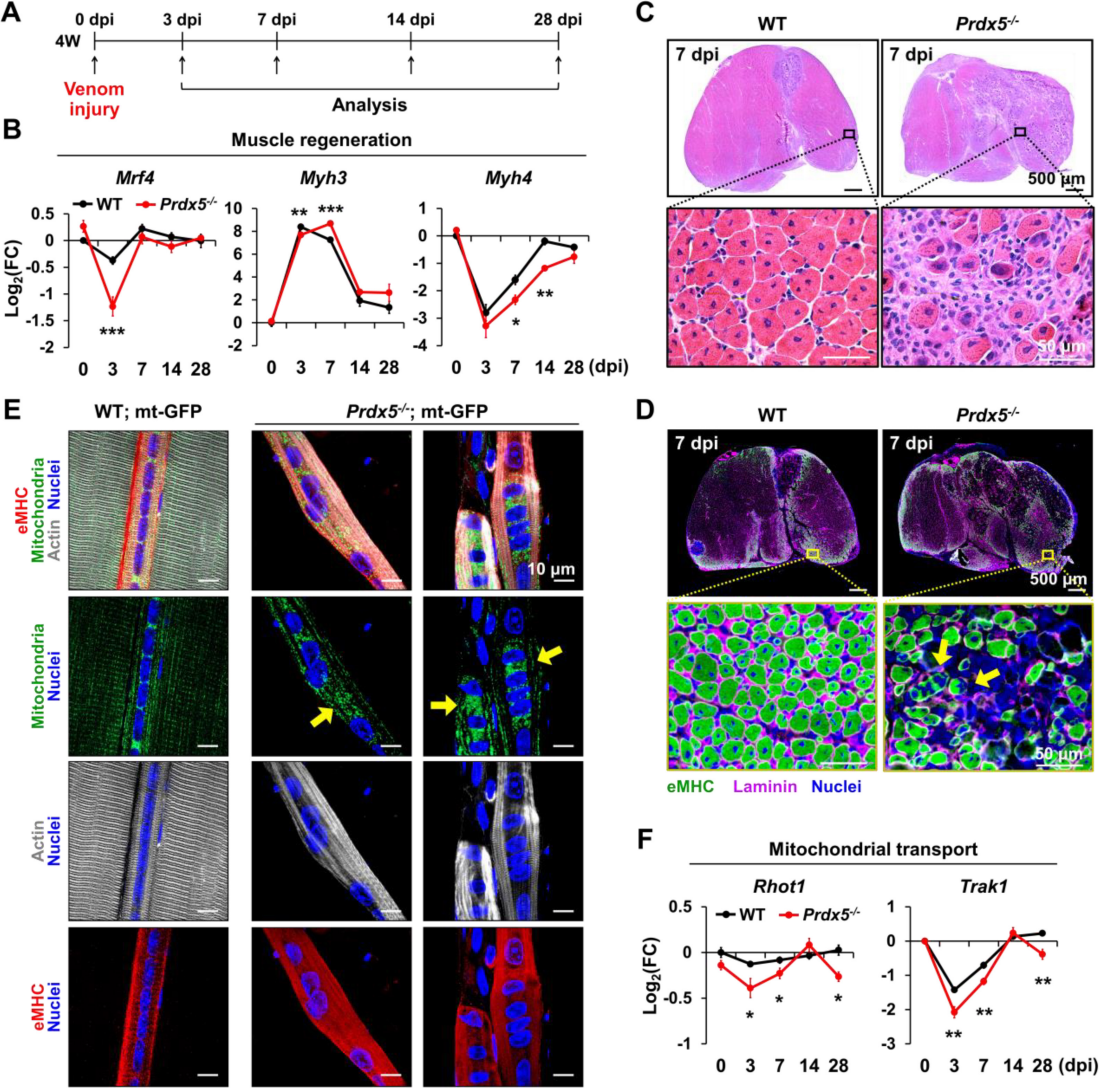
**PRDX5 deficiency impairs muscle regeneration and mitochondrial transport following muscle injury.** (A) Muscle regeneration analysis scheme. Snake venom was locally injected into the GAS and TA muscles of 4‐week‐old mice. (B) qRT‐PCR analysis of muscle regeneration markers in the TA muscles of WT and *Prdx5* ^*−/−*^ mice at 0, 3, 7, 14 and 28 dpi (*n* = 3–7).(C, D) Representative H&E images (C) and fluorescence images (D) of cross‐sections of GAS muscles from WT and *Prdx5* ^*−/−*^ mice at 7 dpi (*n* = 4–7). Magnified images of the boxed regions, shown in the lower panels, display regenerating myofibers characterized by centrally located nuclei or positive staining for eMHC. Note the impaired muscle regeneration in *Prdx5* ^*−/−*^ mice. Arrows in (D) highlight regenerating myofibers with aggregated nuclei in *Prdx5* ^*−/−*^ mice. (E) Representative confocal images of whole‐mount EDL muscles of WT; mt‐GFP and *Prdx5* ^*−/−*^; mt‐GFP mice at 5 dpi. Regenerating myofibers were labelled with anti–eMHC antibody. Arrows highlight abnormal mitochondrial morphology (swollen or fragmented) and alignment in *Prdx5* ^*−/−*^ myofibers. (F) qRT‐PCR analysis of mitochondrial transport regulators in the TA muscles of WT and *Prdx5* ^*−/−*^ mice at 0, 3, 7, 14 and 28 dpi (*n* = 3–7). All scale bars are displayed with actual size values. All data represent mean ± SEM Statistical significance is indicated as *p < 0.05, **p < 0.01 and ***p < 0.001, analysed by *t* test. eMHC, embryonic myosin heavy chain; GAS, gastrocnemius; H&E, haematoxylin and eosin; TA, tibialis anterior.

To investigate the impact of PRDX5 deficiency on muscle regeneration in vivo, we injected the GAS and TA muscles of WT or *Prdx5* ^*−/−*^ mice with snake venom and analysed muscle tissues at 3, 7, 14 and 28 dpi (Figure 6A). Analysis of muscle regeneration marker expression revealed impaired muscle regeneration in *Prdx5* ^*−/−*^ mice, with significantly decreased *Mrf4* and *Myh3* expression at 3 dpi and reduced *Myh4* expression at 7 and 14 dpi (Figure 6B). The delayed maturation of regenerating myofibers in *Prdx5* ^*−/−*^ muscles was further evidenced by a delayed reduction in *Myh3*, which encodes embryonic myosin heavy chain (eMHC), at 7 dpi (Figure 6B). Consistent with the gene expression data, histological analysis at 3 and 7 dpi demonstrated impaired muscle regeneration, as indicated by the defective formation of healthy, centrally nucleated regenerating myofibers (Figure 6C, D and Figure S4A, B). Notably, *Prdx5* ^*−/−*^ mice exhibited regenerating myofibers with aggregated nuclei, similar to what was observed during in vitro myogenesis (Figure 6D). At 28 dpi, histology further revealed persistent defects in regeneration in *Prdx5* ^*−/−*^ muscles, characterized by persistent regions of fibrosis and calcification (Figure S4C). To further examine regenerating myofibers in three dimensions, we performed whole‐mount staining of venom‐injured EDL muscles (Figure S4D). Unlike in WT mice, regenerating myofibers (eMHC‐positive) in *Prdx5* ^*−/−*^ mice exhibited disrupted nuclear distribution and abnormal mitochondrial morphology, characterized by a fragmented or swollen appearance (Figure 6E). Gene expression analysis revealed that *Rhot1* and *Trak1* expression levels were significantly decreased in regenerating muscle tissues of *Prdx5* ^*−/−*^ mice at 3, 7 and 28 dpi, indicating impaired mitochondrial transport (Figure 6F). These findings demonstrate that PRDX5 promotes muscle regeneration in vivo by facilitating proper mitochondrial transport and nuclear distribution.

### 3.6 Combined Deficiency of PRDX3 and PRDX5 Accelerates Muscle Aging Characteristics

**FIGURE 7:**
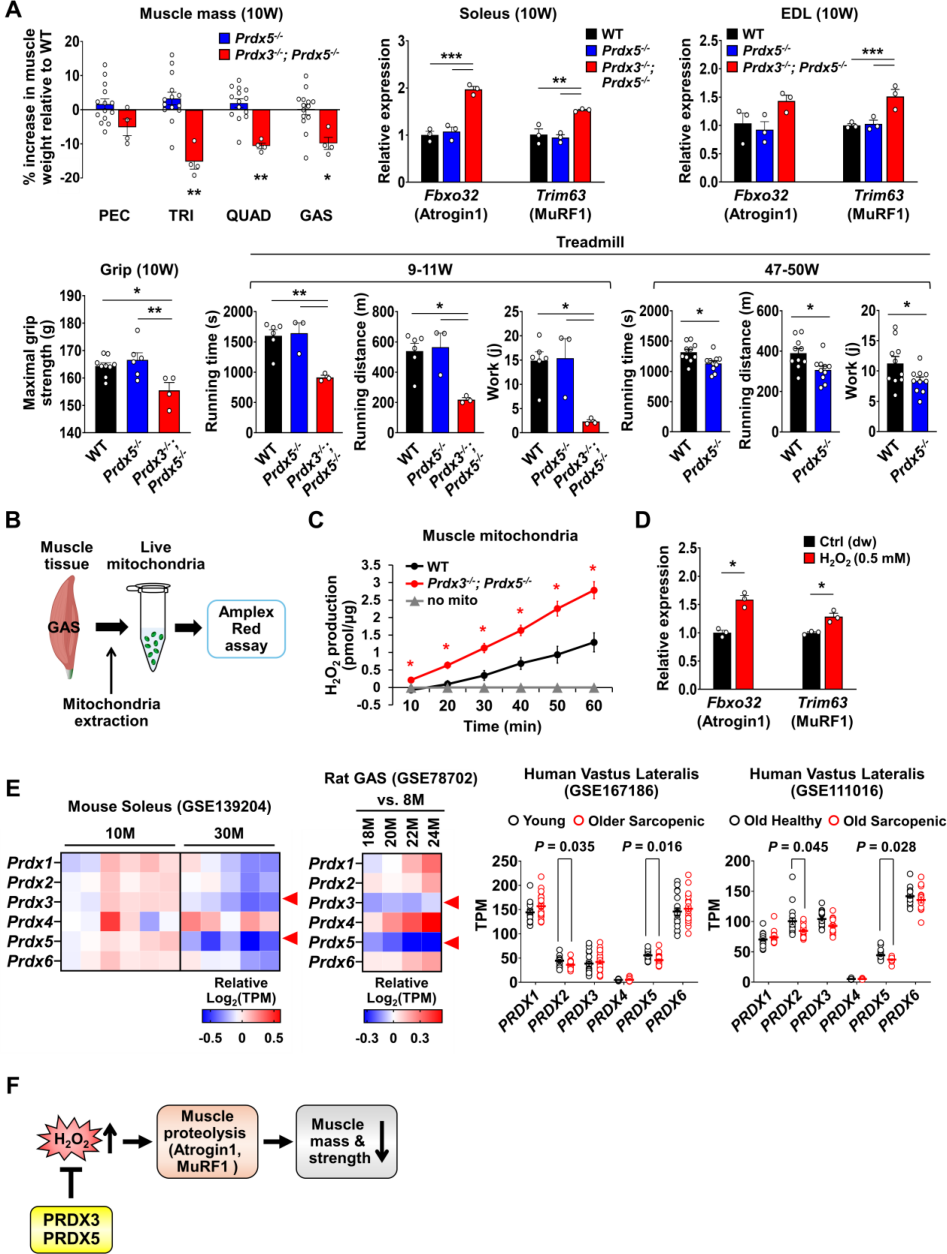
Combined deficiency of PRDX3 and PRDX5 leads to premature muscle aging, characterized by decreased muscle mass and strength, along with increased muscle proteolysis. (A) The leftmost graph in the top row shows muscle weight analysis of 10‐week‐old WT, *Prdx5* ^*−/−*^ and *Prdx3* ^*−/−*^; *Prdx5* ^*−/−*^ mice (*n* = 4–14). The percent increase in muscle weight is presented relative to WT values. PEC, pectoralis; TRI, triceps; QUAD, quadriceps; GAS, gastrocnemius. The two graphs to the right display qRT‐PCR analysis of muscle proteolysis markers *Fbxo32* and *Trim63* in the soleus and EDL muscle tissues of 10‐week‐old WT, *Prdx5* ^*−/−*^ and *Prdx3* ^*−/−*^; *Prdx5* ^*−/−*^ mice (*n* = 3). The leftmost graph in the bottom row presents maximal grip strength (grams, g) analysis of 10‐week‐old WT, *Prdx5* ^*−/−*^ and *Prdx3* ^*−/−*^; *Prdx5* ^*−/−*^ mice (*n* = 4–9). The remaining graphs show treadmill running performance of 9–11‐week‐old WT, *Prdx5* ^*−/−*^ and *Prdx3* ^*−/−*^; *Prdx5* ^*−/−*^ mice (*n* = 3–6), as well as 47–50‐week‐old WT and *Prdx5* ^*−/−*^ mice (*n* = 10). Running time was measured in seconds (s), running distance in meters (m) and work in joules (j). (B, C) Schematic (B) and quantitative analysis (C) of mitochondrial H_2_O_2_ generation using the Amplex Red assay, performed on live mitochondria isolated from GAS muscle tissues of 9–10‐week‐old mice (*n* = 3). Note that the magnitude and rate of H_2_O_2_ production are higher in *Prdx3* ^*−/−*^; *Prdx5* ^*−/−*^ mitochondria than WT mitochondria. (D) qRT‐PCR analysis of muscle proteolysis markers in 48 h myotubes treated with dw (distilled water) or 0.5 mM H_2_O_2_, 24 h after myogenic induction (*n* = 3).(E) Gene expression analysis of *Prdx*/*PRDX* members in skeletal muscle tissues from mice, rats and humans using publicly available transcriptomic datasets. Heatmaps on the left show relative *Prdx* expression in the soleus muscles of 10‐ and 30‐month‐old mice (GSE139204; SarcoAtlas) and the GAS muscles of rats aged 18–24 months compared to 8 months (GSE78702; SarcoAtlas). Red arrowheads indicate marked reductions in *Prdx3* and *Prdx5* expression with aging. Graphs on the right display *PRDX* expression levels in human vastus lateralis muscles from young and older sarcopenic individuals (GSE167186) and from old healthy versus old sarcopenic individuals (GSE111016). Among the *PRDX* genes, PRDX5 shows the most significant downregulation in sarcopenic human muscle, with the lowest *p* value. TPM, transcripts per million.(F) Summary diagram illustrating that PRDX3 and PRDX5 together prevent excessive H_2_O_2_ accumulation, thereby mitigating H_2_O_2_‐induced muscle proteolysis and reducing the risk of muscle atrophy and strength loss. All data represent mean ± SEM Statistical significance is indicated as *p < 0.05, **p < 0.01 and ***p < 0.001, analysed by ANOVA with Tukey's post hoc test (A), or by *t* test (A, C, D). For (E), statistical significance was assessed using the Wald test with Benjamini–Hochberg correction in DESeq2.

We next focused on the analysis of *Prdx3* ^*−/−*^; *Prdx5* ^*−/−*^ mice and found that, unlike *Prdx5* ^*−/−*^ mice, they exhibited significant reductions in muscle mass throughout the body as early as 10 weeks of age (Figure 7A). This muscle mass loss was accompanied by an increase in *Fbxo32* and *Trim63* expression, which encode the muscle‐specific E3 ubiquitin ligases Atrogin1 and MURF1, respectively, in the soleus and EDL muscles of *Prdx3* ^*−/−*^; *Prdx5* ^*−/−*^ mice at 10 weeks of age (Figure 7A). In addition to reduced muscle mass, grip strength and treadmill running performance were significantly decreased in *Prdx3* ^*−/−*^; *Prdx5* ^*−/−*^ mice compared to WT or *Prdx5* ^*−/−*^ mice at 9–11 weeks of age (Figure 7A). The muscle atrophy and functional reduction observed in *Prdx3* ^*−/−*^; *Prdx5* ^*−/−*^ mice at a young age reflect a sarcopenic phenotype, suggesting that the combined deficiency of PRDX3 and PRDX5 accelerates muscle aging. While *Prdx5* ^*−/−*^ mice did not exhibit muscle mass or strength loss at a young age, their running performance was significantly impaired at 47–50 weeks of age, reflecting mitochondrial dysfunction in muscle (Figure 7A). Fat mass was also significantly increased in *Prdx5* ^*−/−*^ mice at 47–50 weeks of age (Figure S5).

To examine whether excessive H_2_O_2_ accumulation drives the muscle aging phenotype observed in *Prdx3* ^*−/−*^; *Prdx5* ^*−/−*^ mice, we isolated live mitochondria from muscle tissues and performed Amplex Red assay to measure H_2_O_2_ production levels (Figure 7B). Compared to mitochondria from WT muscle, those from *Prdx3* ^*−/−*^; *Prdx5* ^*−/−*^ muscle showed a significant increase in H_2_O_2_ production (Figure 7C). Furthermore, H_2_O_2_ treatment of myotubes significantly upregulated the expression of muscle proteolysis markers *Fbxo32* and *Trim63* (Figure 7D), demonstrating that excessive H_2_O_2_ accumulation contributes to the muscle aging phenotype in *Prdx3* ^*−/−*^; *Prdx5* ^*−/−*^ mice. To investigate additional mechanisms driving Atrogin1 and MURF1 expression beyond elevated oxidative stress, we evaluated the expression of *Ppargc1a* (PGC‐1α), a known suppressor of Atrogin1 and MURF1 transcription [33] (Figure S6A). *Ppargc1a* expression was significantly reduced in *Prdx3* ^*−/−*^ *; Prdx5* ^*−/−*^ muscles, supporting the possibility that its downregulation contributes to the elevated expression of muscle proteolysis genes in the double knockout muscles. However, *Ppargc1a* expression was also significantly decreased in the soleus of *Prdx5* ^*−/−*^ mice at 10 weeks of age, despite no significant increase in Atrogin1 or MuRF1 levels (Figure 7A and Figure S6A). This suggests that a threshold level of PGC‐1α may exist, below which the induction of these proteolytic genes is triggered.

Next, we analysed publicly available datasets to assess changes in *Prdx* gene expression during aging and under pathological muscle conditions. Among the *Prdx* genes, *Prdx3* and *Prdx5* were the two most dramatically downregulated genes in aged mouse soleus and aged rat GAS (Figure 7E). Notably, *Prdx3* and *Prdx5* were the only downregulated *Prdx* genes in aged rat GAS, whereas all other *Prdx* genes were upregulated at 22 and 24 months of age compared to 8 months of age (Figure 7E), highlighting a strong association between muscle aging and reduced PRDX3 and PRDX5 levels in rodent muscles. *Prdx3* and *Prdx5* levels also showed a decreasing trend in mouse models of Duchenne muscular dystrophy (DMD) and dysferlinopathy‐associated accelerated muscle degeneration (Figure S6B). *Trak1* expression was also significantly reduced in aged mouse soleus and GAS, mirroring the expression pattern of *Prdx5* (Figure S6C). In human vastus lateralis muscle, *PRDX2* and *PRDX5* expression levels were significantly decreased in sarcopenic individuals compared to young or age‐matched healthy individuals. Among them, *PRDX5* showed the most significant decrease, highlighting its potential importance in human muscle aging (Figure 7E). Collectively, these findings suggest that PRDX3 and PRDX5 work together to prevent excessive H_2_O_2_ accumulation, thereby mitigating muscle proteolysis, muscle atrophy and strength loss, and ultimately preserving both muscle quantity and quality, and that their loss may lead to accelerated muscle aging (Figure 7F).

## 4 Discussion

In this study, we identified a previously unrecognized role of PRDX5 in skeletal muscle, which involves maintaining mitochondrial function and ensuring proper nuclear distribution during myogenesis and muscle regeneration. Using *Prdx3* ^*−/−*^, *Prdx5* ^*−/−*^ and *Prdx3* ^*−/−*^; *Prdx5* ^*−/−*^ mice, we demonstrated that PRDX5 deficiency, but not PRDX3 deficiency, disrupts myonuclear spreading during myotube formation, indicating that PRDX5 is the primary regulator of nuclear distribution. Mechanistically, we found that PRDX5 supports mitochondrial ATP production and induces the expression of mitochondrial transport regulator genes *Rhot1* (encoding Miro1) and *Trak1* (encoding Milton), both of which are essential for effective mitochondrial transport and proper myonuclear spreading during muscle development and regeneration. Moreover, we observed that *Prdx3* ^*−/−*^; *Prdx5* ^*−/−*^ mice exhibited an accelerated muscle aging phenotype, characterized by significantly reduced muscle mass and strength as well as increased muscle protein degradation as early as 10 weeks of age. This phenotype was associated with elevated oxidative stress, as muscle mitochondria from *Prdx3* ^*−/−*^; *Prdx5* ^*−/−*^ mice produced significantly higher levels of H_2_O_2_ compared to those from WT mice. This increased oxidative stress upregulated muscle‐specific E3 ligases, Atrogin1 and MuRF1, which are known to promote muscle atrophy, suggesting that PRDX3 and PRDX5 function cooperatively to preserve muscle mass.

One of the most significant findings of our study is that PRDX5 regulates myonuclear distribution by promoting the expression of *Rhot1* and *Trak1*. siRNA‐mediated downregulation of these genes impaired mitochondrial transport and led to increased nuclear clustering in myotubes, highlighting that proper mitochondrial transport, facilitated by PRDX5, is essential for myonuclear spreading. However, the mechanism by which PRDX5 regulates the transcription of these genes remains to be elucidated. While PRDXs are well‐known for their antioxidant functions, they also perform antioxidant‐independent activities such as directly modulating transcription factors. For instance, a previous study demonstrated that PRDX5 can directly interact with heterogeneous nuclear ribonucleoprotein K (hnRNPK) in the nucleus to regulate gene expression in osteoblasts [22]. Interestingly, dysregulated hnRNPK activity has been reported to cause the formation of abnormal myotubes with locally spherical morphology [34], which is similar to the phenotype observed in many PRDX5‐deficient myotubes, implying that hnRNPK may play a significant role in the actions of PRDX5 during myotube formation. PRDX5 has also been reported to directly bind to nuclear factor erythroid 2‐related factor 2 (NRF2), a key transcription factor that binds to antioxidant response element (ARE) sites to enhance the expression of various cytoprotective genes [21]. These findings suggest that PRDX5 may regulate the expression of *Rhot1* and *Trak1* through similar mechanisms, possibly by directly modulating the activity of transcription factors specific to *Rhot1* and *Trak1*. Notably, we performed transcription factor binding site analysis using publicly available databases, such as Tfsitescan and GeneHancer [35, 36], and identified specificity protein 1 (SP1) as one of the top transcription factors for both *Rhot1* and *Trak1*. SP1 has been shown to form a complex with NRF2 [37], suggesting that PRDX5 may potentially regulate *Rhot1* and *Trak1* expression by modulating SP1. However, transcription factors for *Rhot1* and *Trak1* have not been directly tested in experiments to date, and future studies will investigate whether PRDX5 modulates transcription factor activities to promote *Rhot1* and *Trak1* expression. Further investigation is also needed to clarify the mechanistic link between mitochondrial distribution and nuclear spreading. One plausible explanation is that proper mitochondrial positioning ensures localized ATP supply, which is crucial for kinesin‐mediated nuclear movement that requires ATP [38].

Both mitochondria and nuclei are transported along the microtubule cytoskeleton during myogenesis [17, 39]. In our analysis, a subset of PRDX5‐deficient myotubes displayed disrupted microtubule networks (Movie 3 and Movie 5), suggesting that defective microtubule structures may contribute to the impaired mitochondrial and nuclear distribution observed in these myotubes. However, it remains unclear whether this disruption is a cause or a consequence of impaired myogenesis in PRDX5‐deficient myotubes. Notably, some PRDX5‐deficient myotubes with impaired nuclear spreading did not display significant microtubule defects, complicating the interpretation of these results. Similarly, a previous study reported that microtubule‐associated protein 7 (MAP 7)‐depleted myotubes exhibited disrupted nuclear spreading without noticeable microtubule defects [16], indicating that microtubule integrity alone may not fully explain the observed phenotypes. Further research is needed to determine whether PRDX5 directly influences microtubule stability and organization during myotube formation.

Unlike *Prdx3* ^*−/−*^; *Prdx5* ^*−/−*^ mice, which exhibited significant reductions in muscle mass, PRDX5 deficiency alone did not significantly affect muscle mass, particularly at a young age, suggesting that intracellular H_2_O_2_ levels in *Prdx5* ^*−/−*^ muscles may not have reached the threshold required to induce muscle wasting. It is possible that compensatory actions by other PRDX isoforms, glutathione peroxidases, or catalases that regulate H_2_O_2_ levels may have mitigated oxidative stress in the absence of PRDX5. However, prolonged PRDX5 deficiency eventually led to muscle mitochondrial dysfunction, compromising muscle quality and running endurance capacity. Although dysregulated H_2_O_2_ levels likely have directly contributed to the muscle defects observed in *Prdx5* ^*−/−*^ mice, our results suggest that impaired mitochondrial ATP production and transport, along with the resultant defects in myonuclear spreading, may be the major drivers of reduced exercise capacity and impaired muscle regeneration in these mice. Lean mass, as measured by DXA, did not significantly decrease even at 47–50 weeks of age in *Prdx5* ^*−/−*^ mice, indicating that PRDX5 is more critical for preserving muscle quality rather than muscle quantity. Importantly, the combined deficiency of PRDX3 and PRDX5 led to substantial muscle loss at a young age, highlighting their cooperative roles in preserving muscle mass. The accelerated muscle atrophy observed in *Prdx3* ^*−/−*^; *Prdx5* ^*−/−*^ mice suggests that PRDX3 and PRDX5 work synergistically to prevent excessive oxidative damage and maintain both muscle mass and strength. These findings underscore the importance of antioxidant defences in preventing age‐related muscle degeneration and highlight the potential of augmenting PRDX3 and PRDX5 activity as a therapeutic strategy to combat sarcopenia and muscle aging.

In summary, our study revealed that PRDX5 is a crucial regulator of mitochondrial function and nuclear distribution during myogenesis and muscle regeneration. We demonstrated that PRDX5 promotes proper myonuclear spreading by upregulating Miro1 and Milton, which are critical for effective mitochondrial transport. Additionally, the combined deficiency of PRDX3 and PRDX5 accelerated muscle aging by exacerbating oxidative stress and mitochondrial dysfunction. These findings provide a deeper understanding of the mechanisms by which PRDX5 preserves skeletal muscle integrity and suggest that enhancing the expression or activity of PRDX5 and PRDX3 could serve as a promising therapeutic strategy to counteract sarcopenia and age‐associated muscle degeneration.

## Conflicts of Interest

The authors declare no conflicts of interest.

## Supporting information


**Movie 1** Nuclear and mitochondrial distribution during in vitro myogenesis. Time‐lapse movie showing the differentiation of WT; mt‐GFP myoblasts into myotubes over 15 h. The arrow highlights nuclear centration followed by nuclear spreading.

**Movie 2** Impaired nuclear and mitochondrial spreading in *Prdx5*‐knockout myotubes. Time‐lapse movie showing the differentiation of WT; mt‐GFP and *Prdx5* ^*−/−*^; mt‐GFP myoblasts into myotubes over 22 h. Myoblasts were transfected with a Lifeact plasmid to visualize the actin cytoskeleton in red. The arrow in WT; mt‐GFP indicates the actin‐labelled myotube.

**Movie 3** Super‐resolution imaging of normal and *Prdx5*‐knockout myotubes. Time‐lapse movie captured using Zeiss Elyra 7 with Lattice SIM, showing the differentiation of WT; mt‐GFP and *Prdx5* ^*−/−*^; mt‐GFP myoblasts into myotubes over 340 min. Myoblasts were transfected with a *Map 7* plasmid to visualize microtubules in red. Hoechst was used to stain nuclei in blue. Nuclear and mitochondrial spreading are impaired in *Prdx5* ^*−/−*^; mt‐GFP myotubes.

**Movie 4** Decreased ATP production and abnormal mitochondrial morphology in *Prdx5*‐deficient myotubes. Short‐term continuous live imaging captured using a confocal microscope in Airyscan mode, showing developing WT; mt‐GFP, *Prdx5* ^*−/−*^; mt‐GFP and *Prdx3* ^*−/−*^; *Prdx5* ^*−/−*^; mt‐GFP myotubes at 24 h of differentiation. Myotubes were stained with ATP‐Red dye to visualize mitochondrial ATP in red. Hoechst was used to stain nuclei in blue.

**Movie 5** Super‐resolution imaging of mitochondrial transport in normal and *Prdx5*‐ or *Trak1*‐deficient myotubes. Short‐term continuous live imaging captured using Zeiss Lattice SIM 5, showing 48‐h WT; mt‐GFP myotubes transfected with siCtrl, si*Prdx5*, or si*Trak1*. Myotubes were also transfected with a *Map 7* plasmid to visualize microtubules in red. Hoechst was used to stain nuclei in blue. Mitochondrial transport along the microtubules is impaired in myotubes deficient in *Prdx5* or *Trak1*.

**Figure S1:** jcsm70098‐sup‐0006‐Supplementary_Material.docx. *Prdx5* deficiency tends to induce mitochondrial fragmentation in myotubes. (A, B) qRT‐PCR analysis of mitochondrial fusion markers (A) and fission markers (B) in 48 h myotubes (*n* = 3). The absence of *Prdx5* results in a significantly decreased expression of *Mfn2*, a mitochondrial fusion marker. All data represent mean ± SEM, analysed by ANOVA with Tukey's post hoc test.
**Figure S2:** Impaired ATP production increases myonuclear clustering. (A, B) Representative confocal images (A) and quantitative analysis (B) of myotubes treated with Oligomycin A, a mitochondrial ATP synthase inhibitor (*n* = 3 independent wells; 110–116 myotubes). Oligomycin A (0.1 μM) was administered to developing myotubes at 24 h and analysed at 48 h. The percentage of myotubes containing more than five clustered nuclei was measured in (B). Data represent mean ± SEM. Statistical significance is indicated as ***p < 0.001, analysed by *t* test.
**Figure S3:** Gene expression changes in myotubes following modulation of Rhot1 and Trak1 expression. (A) qRT‐PCR analysis of *Rhot1* and *Trak1* expression in myotubes treated with distilled water (dw) or 0.5 mM H_2_O_2_, 24 h after myogenic induction and collected at 48 h (*n* = 3). (B) qRT‐PCR analysis of motor function‐related genes in WT, *Prdx5* ^*−/−*^ and *Prdx3* ^*−/−*^; *Prdx5* ^*−/−*^ myotubes at 48 h (*n* = 3). (C) qRT‐PCR analysis of gene knockdown efficiency following siRNA treatment (*n* = 3).(D) qRT‐PCR analysis of myogenic markers in siRNA‐treated myotubes at 48 h (*n* = 3).(E) qRT‐PCR analysis of *Rhot1* and *Trak1* expression following overexpression of each gene alone or in combination in myotubes at 48 h (*n* = 3). Data represent mean ± SEM. Statistical significance is indicated as *p < 0.05, **p < 0.01 and ***p < 0.001, analysed by ANOVA with Tukey's post hoc test (A, D, E), or by *t* test (B, C).
**Figure S4: Muscle regeneration at 3 and 28 days after injury (dpi).**(A, B) Representative fluorescence images (A) and haematoxylin and eosin (H&E) images (B) of cross‐sections of gastrocnemius (GAS) muscles from WT and *Prdx5* ^*−/−*^ mice at 3 dpi. Magnified images of the boxed regions, shown in the lower panels in (A), display regenerating myofibers characterized by centrally located nuclei or positive staining for embryonic myosin heavy chain (eMHC). (C) H&E images of GAS muscle cross‐sections from WT and *Prdx5* ^*−/−*^ mice at 28 dpi. Lower panels show magnified views of boxed regions 1 and 2.(D) Fluorescence images of extensor digitorum longus (EDL) muscle cross‐sections from WT mice at 5 dpi. Venom was injected into the tibialis anterior (TA), which also damages the underlying EDL. Staining for eMHC confirms activation of regenerative processes in the EDL following TA injury. All scale bars are displayed with actual size values.
**Figure S5: Prdx5 deficiency results in increased fat mass.** (A–D) Representative dual X‐ray absorptiometry (DXA) images (A) and corresponding analysis (B–D) of 47‐ to 50‐week‐old WT and *Prdx5* ^*−/−*^ mice (*n* = 6–7). Total fat mass was significantly increased in *Prdx5* ^*−/−*^ mice (C). Scale bars are displayed with actual size value. All data represent mean ± SEM. Statistical significance is indicated as **p < 0.01, analysed by *t* test.
**Figure S6: Prdx‐related gene expression in aging and muscle pathology models.** (A) qRT‐PCR analysis of *Ppargc1a* expression in soleus and EDL muscles from WT, *Prdx5* ^*−/−*^ and *Prdx3* ^*−/−*^; *Prdx5* ^*−/−*^ mice at 10 weeks of age (*n* = 3). Data represent mean ± SEM. Statistical significance is indicated as *p < 0.05, **p < 0.01 and ***p < 0.001, analysed by ANOVA with Tukey's post hoc test. (B) Heatmaps showing relative expression of *Prdx* genes in mouse skeletal muscles using publicly available datasets. Left: quadriceps (QUAD) muscles from WT, *F66* and *F66*; *Dysf* ^*−/−*^ mice (GSE62945; our previously published dataset), a model exhibiting accelerated muscle degeneration associated with dysferlinopathy. Right: TA muscles from WT and *mdx* mice (GSE132741), a model of Duchenne muscular dystrophy (DMD). Red arrowheads indicate marked downregulation of *Prdx3* and *Prdx5*. (C) Expression levels of *Rhot1* and *Trak1* in mouse soleus and GAS muscles at 10 and 30 months of age, obtained from the GSE139204 dataset (SarcoAtlas).
**Table S1:** Genotyping primer sequences.
**Table S2:** qRT‐PCR primer sequences.
