# Supplementary material for: PRDX5 Regulates Mitochondrial Function and Nuclear Spreading in Myogenesis and Acts With PRDX3 to Delay Muscle Aging
Source: J Cachexia Sarcopenia Muscle. 2025 Oct 28;16(6):e70098. doi: 10.1002/jcsm.70098 (PMC12559808; doi:10.1002/jcsm.70098)
Supplement: Supplementary file 6 — Figure S1: jcsm70098‐sup‐0006‐Supplementary_Material.docx. Prdx5 deficiency tends to induce mitochondrial fragmentation in myotubes. (A, B) qRT‐PCR analysis of mitochondrial fusion markers (A) and fission markers (B) in 48 h myotubes (n = 3). The absence of Prdx5 results in a significantly decreased expression of Mfn2, a mitochondrial fusion marker. All data represent mean ± SEM, analysed by ANOVA with Tukey's post hoc test. Figure S2: Impaired ATP production increases myonuclear clustering. (A, B) Representative confocal images (A) and quantitative analysis (B) of myotubes treated with Oligomycin A, a mitochondrial ATP synthase inhibitor (n = 3 independent wells; 110–116 myotubes). Oligomycin A (0.1 μM) was administered to developing myotubes at 24 h and analysed at 48 h. The percentage of myotubes containing more than five clustered nuclei was measured in (B). Data represent mean ± SEM. Statistical significance is indicated as ***p < 0.001, analysed by t test. Figure S3: Gene expression changes in myotubes following modulation of Rhot1 and Trak1 expression. (A) qRT‐PCR analysis of Rhot1 and Trak1 expression in myotubes treated with distilled water (dw) or 0.5 mM H2O2, 24 h after myogenic induction and collected at 48 h (n = 3). (B) qRT‐PCR analysis of motor function‐related genes in WT, Prdx5 −/− and Prdx3 −/− ; Prdx5 −/− myotubes at 48 h (n = 3). (C) qRT‐PCR analysis of gene knockdown efficiency following siRNA treatment (n = 3).(D) qRT‐PCR analysis of myogenic markers in siRNA‐treated myotubes at 48 h (n = 3).(E) qRT‐PCR analysis of Rhot1 and Trak1 expression following overexpression of each gene alone or in combination in myotubes at 48 h (n = 3). Data represent mean ± SEM. Statistical significance is indicated as *p < 0.05, **p < 0.01 and ***p < 0.001, analysed by ANOVA with Tukey's post hoc test (A, D, E), or by t test (B, C). Figure S4: Muscle regeneration at 3 and 28 days after injury (dpi).(A, B) Representative fluorescence images (A) and haematoxylin and [file JCSM-16-e70098-s004.docx]

**SUPPLEMENTARY FIGURES 1-6**


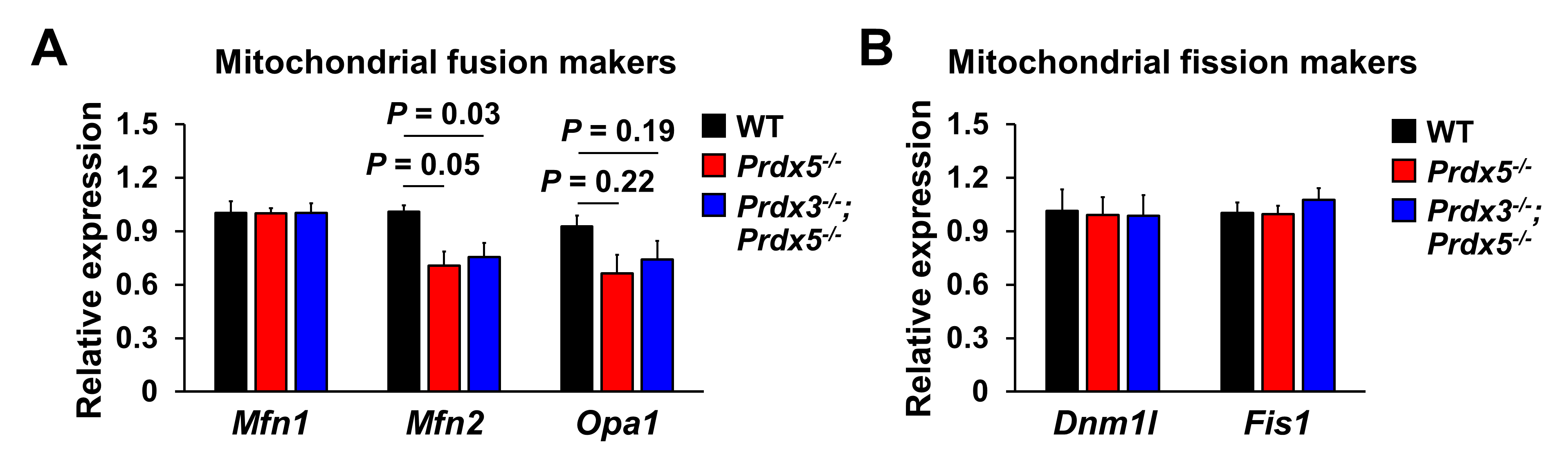


**Figure S1. *Prdx5* deficiency tends to induce mitochondrial fragmentation in myotubes.**

(A, B) qRT-PCR analysis of mitochondrial fusion markers (A) and fission markers (B) in 48 hr myotubes (*n* = 3). The absence of *Prdx5* results in a significantly decreased expression of *Mfn2*, a mitochondrial fusion marker.

All data represent mean ± SEM, analyzed by ANOVA with Tukey’s post hoc test.


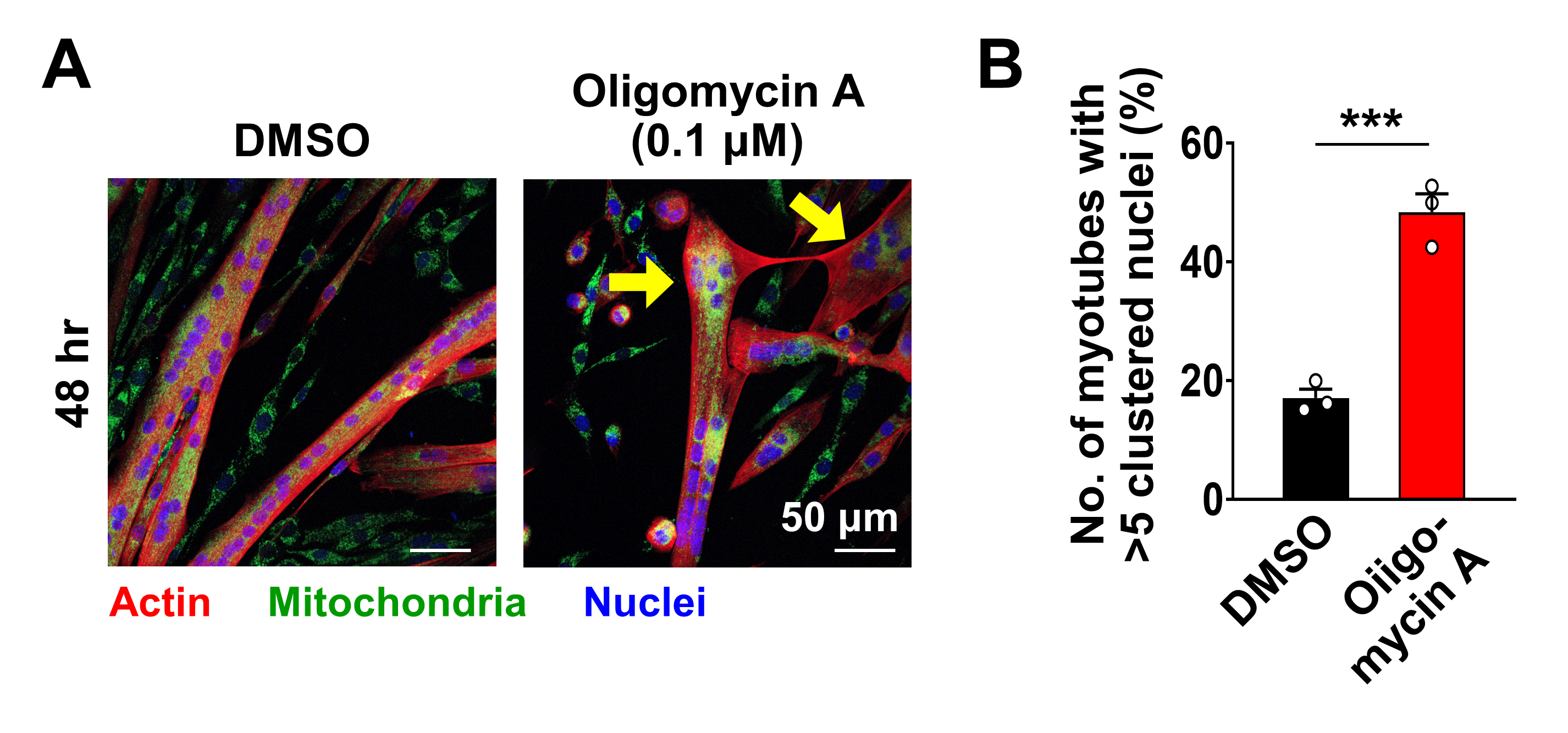


**Figure S2. Impaired ATP production increases myonuclear clustering.**

(A, B) Representative confocal images (A) and quantitative analysis (B) of myotubes treated with Oligomycin A, a mitochondrial ATP synthase inhibitor (*n* = 3 independent wells; 110-116 myotubes). Oligomycin A (0.1 μM) was administered to developing myotubes at 24 hr and analyzed at 48 hr. The percentage of myotubes containing more than 5 clustered nuclei was measured in (B).

Data represent mean ± SEM. Statistical significance is indicated as ****P* < 0.001, analyzed by t test.


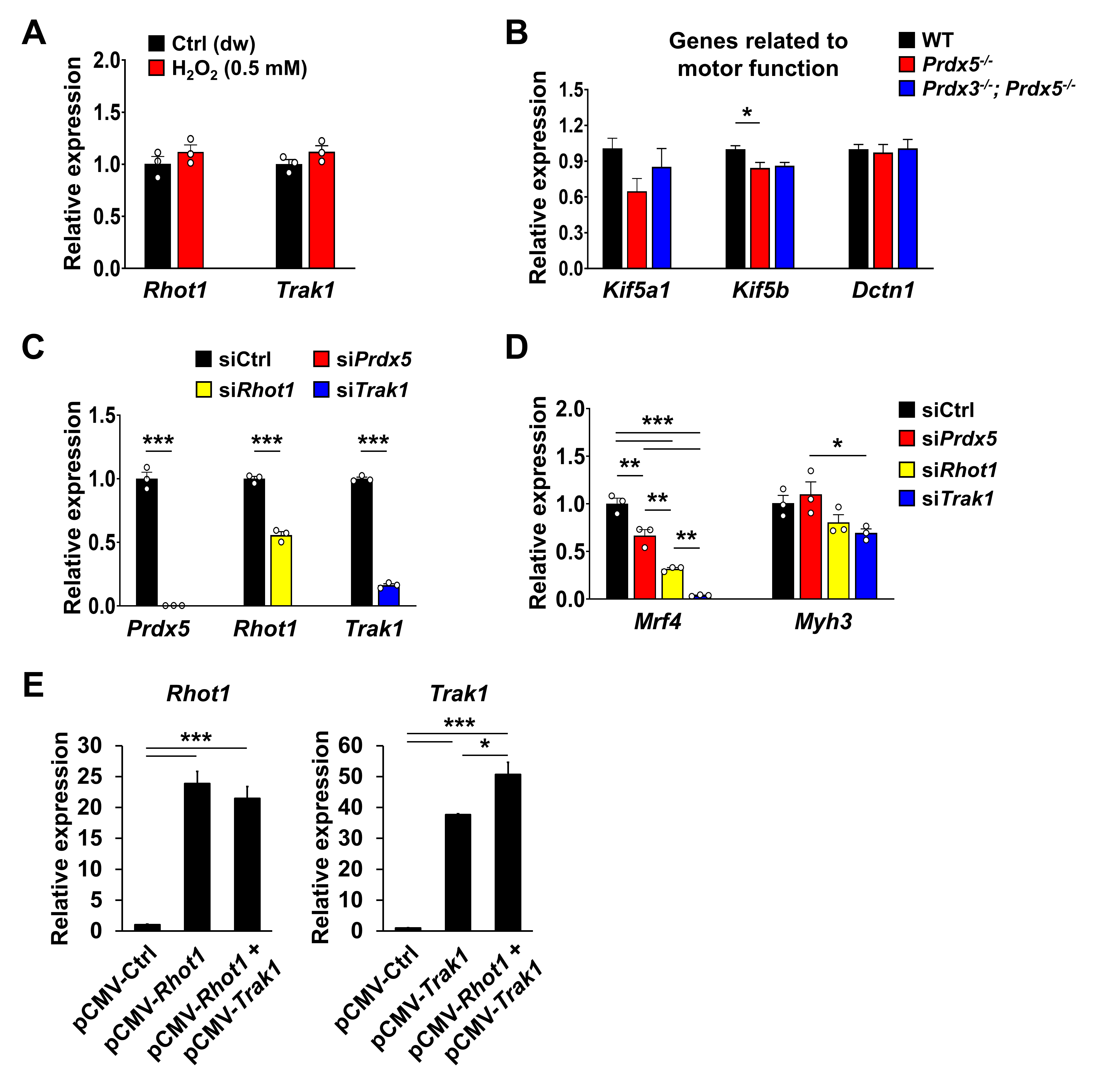


**Figure S3. Gene expression changes in myotubes following modulation of *Rhot1* and *Trak1* expression.**

(A) qRT-PCR analysis of *Rhot1* and *Trak1* expression in myotubes treated with distilled water (dw) or 0.5 mM H_2_O_2_, 24 hours after myogenic induction and collected at 48 hr (*n* = 3).

(B) qRT-PCR analysis of motor function-related genes in WT, *Prdx5^-/-^*, and *Prdx3^-/-^*; *Prdx5^-/-^* myotubes at 48 hr (*n* = 3).

(C) qRT-PCR analysis of gene knockdown efficiency following siRNA treatment (*n* = 3).

(D) qRT-PCR analysis of myogenic markers in siRNA-treated myotubes at 48 hr (*n* = 3).

(E) qRT-PCR analysis of *Rhot1* and *Trak1* expression following overexpression of each gene alone or in combination in myotubes at 48 hr (*n* = 3).

Data represent mean ± SEM. Statistical significance is indicated as **P* < 0.05, ***P* < 0.01, and ****P* < 0.001, analyzed by ANOVA with Tukey’s post hoc test (A, D, E), or by t test (B, C).

**Figure S4. Muscle regeneration at 3 and 28 days post-injury (dpi).**

(A, B) Representative fluorescence images (A) and hematoxylin and eosin (H&E) images (B) of cross-sections of gastrocnemius (GAS) muscles from WT and *Prdx5^-/-^* mice at 3 dpi. Magnified images of the boxed regions, shown in the lower panels in (A), display regenerating myofibers characterized by centrally located nuclei or positive staining for embryonic myosin heavy chain (eMHC).

(C) H&E images of GAS muscle cross-sections from WT and *Prdx5^-/-^* mice at 28 dpi. Lower panels show magnified views of boxed regions 1 and 2.

(D) Fluorescence images of extensor digitorum longus (EDL) muscle cross-sections from WT mice at 5 dpi. Venom was injected into the tibialis anterior (TA), which also damages the underlying EDL. Staining for eMHC confirms activation of regenerative processes in the EDL following TA injury.

All scale bars are displayed with actual size values.


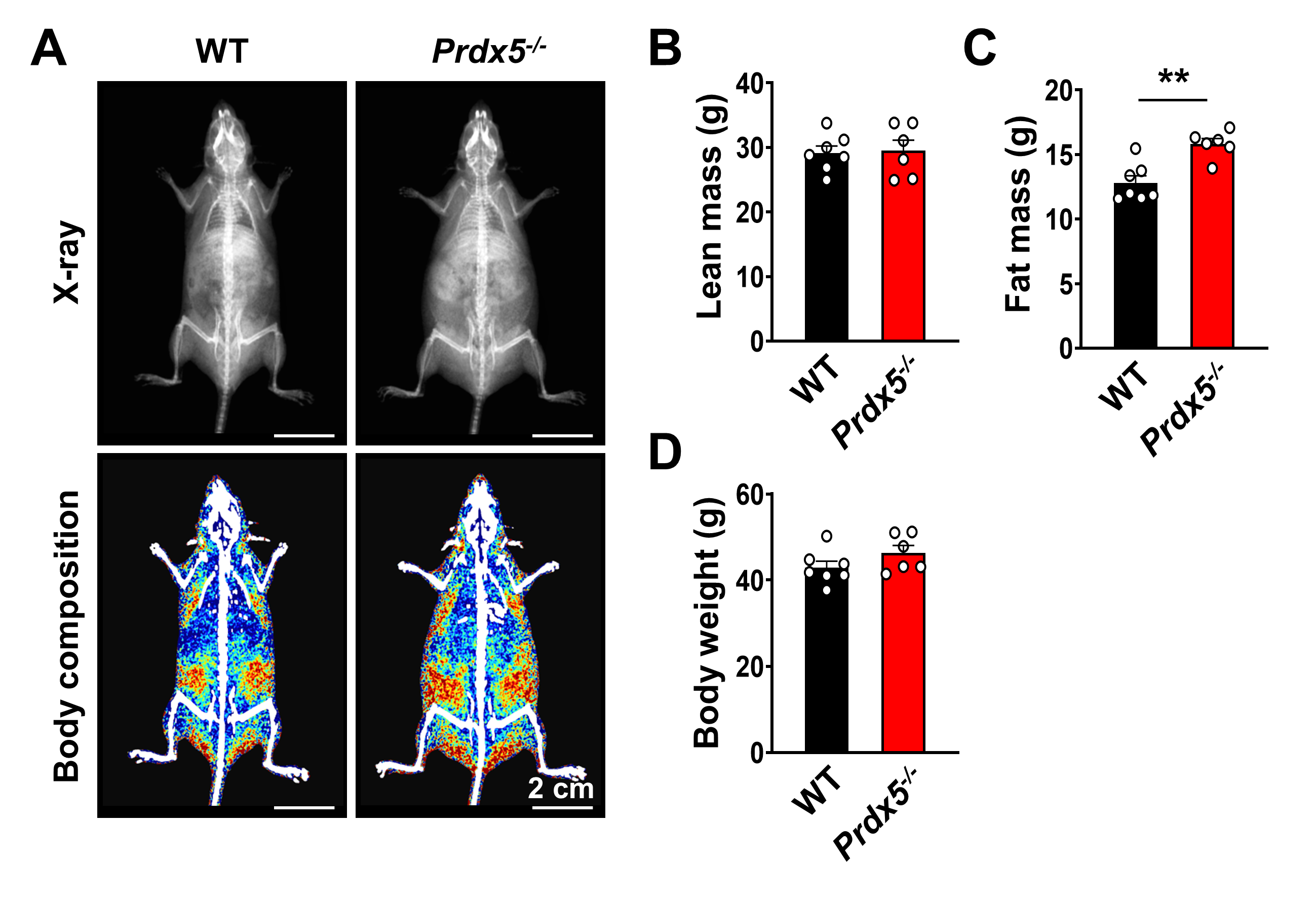


**Figure S5. *Prdx5* deficiency results in increased fat mass.**

(A-D) Representative dual X-ray absorptiometry (DXA) images (A) and corresponding analysis (B-D) of 47- to 50-week-old WT and *Prdx5^-/-^* mice (*n* = 6-7). Total fat mass was significantly increased in *Prdx5^-/-^* mice (C). Scale bars are displayed with actual size value.

All data represent mean ± SEM. Statistical significance is indicated as ***P* < 0.01, analyzed by t test.


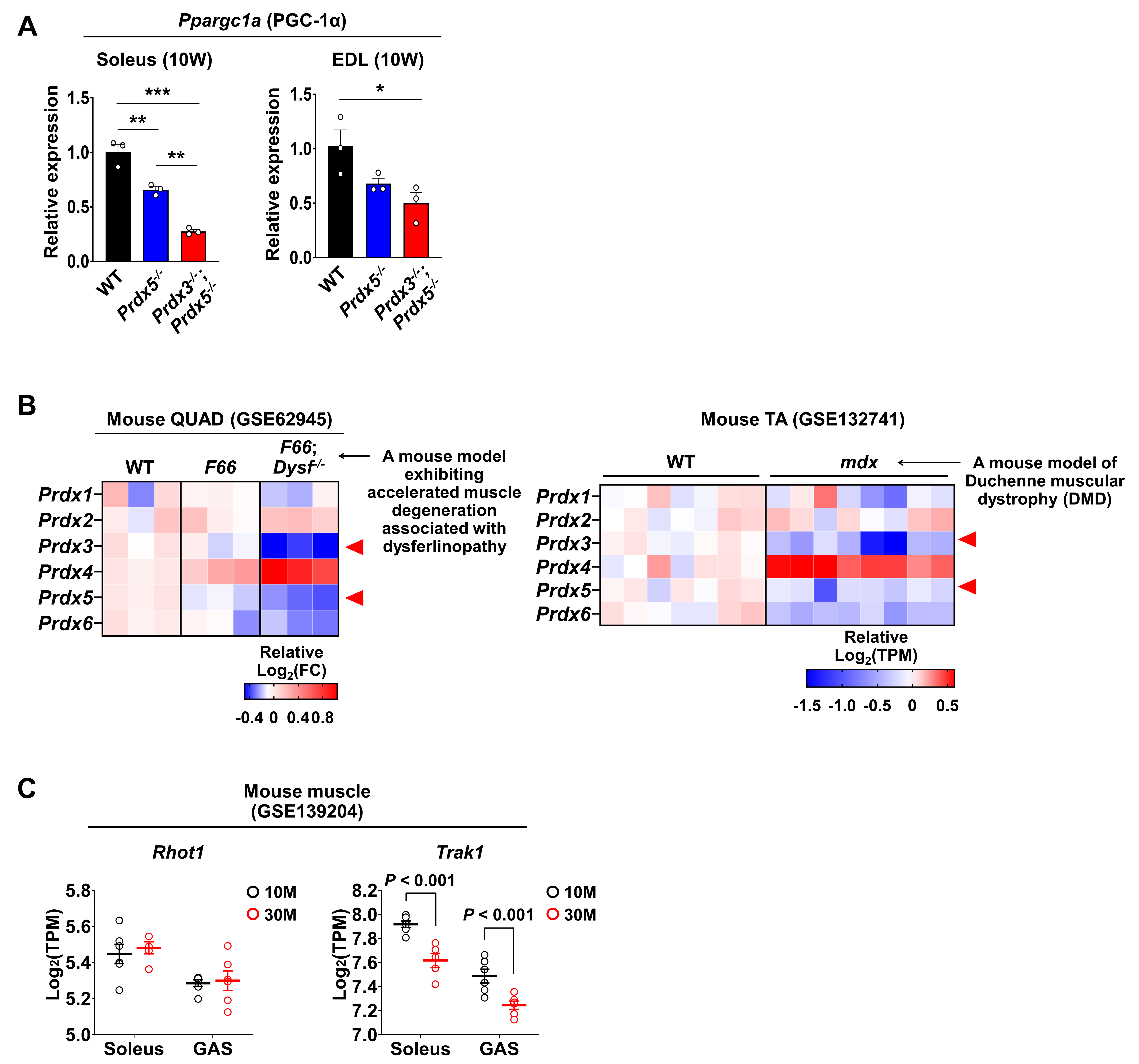


**Figure S6. *Prdx*-related gene expression in aging and muscle pathology models.**

(A) qRT-PCR analysis of *Ppargc1a* expression in soleus and EDL muscles from WT, *Prdx5^-/-^*, and *Prdx3^-/-^*; *Prdx5^-/-^* mice at 10 weeks of age (*n* = 3). Data represent mean ± SEM. Statistical significance is indicated as **P* < 0.05, ***P* < 0.01, and ****P* < 0.001, analyzed by ANOVA with Tukey’s post hoc test.

(B) Heatmaps showing relative expression of *Prdx* genes in mouse skeletal muscles using publicly available datasets. Left: quadriceps (QUAD) muscles from WT, *F66*, and *F66*; *Dysf^-/-^* mice (GSE62945; our previously published dataset), a model exhibiting accelerated muscle degeneration associated with dysferlinopathy. Right: TA muscles from WT and *mdx* mice (GSE132741), a model of Duchenne muscular dystrophy (DMD). Red arrowheads indicate marked downregulation of *Prdx3* and *Prdx5*.

(C) Expression levels of *Rhot1* and *Trak1* in mouse soleus and GAS muscles at 10 and 30 months of age, obtained from the GSE139204 dataset (SarcoAtlas).

| **Table S1. Genotyping primer sequences** | |
| --- | --- |
| Gene | Primer sequence (5’-3’) |
| *Prdx3* | F2: CAGGAAATGTCAATAAGTGTCTAC |
|  | R2: CGAGGACCAGAGCAACCTTC |
|  | neo-R3: CCAGAGGCCACTTGTGTAGC |
| *Prdx5* | Fwd: ATTCTTTGGTGTCTCTCTTTGGG |
|  | Rev: CTTCACTTTCTCCTCCAAATCCC |
|  | KO-Fwd: CCCGTCATATTGCTGAAGAGC |
| mt-GFP | MT26U1: CTCTGCTGCCTCCTGGCTTCT |
|  | MT26U2: CGAGGCGGATCACAAGCAATA |
|  | MT26U3: TCAATGGGCGGGGGTCGTT |

| **Table S2. qRT-PCR primer sequences** | |
| --- | --- |
| Gene | Primer sequence (5’-3’) |
| *Prdx1* | F: TGTCCCACGGAGATCATTGC |
|  | R: GGGTGTGTTAATCCATGCCAG |
| *Prdx2* | F: CACCTGGCGTGGATCAATACC |
|  | R: GACCCCTGTAAGCAATGCCC |
| *Prdx3* | F: AATGGTGGTTTGGGCCACAT |
|  | R: TGACACTCAGGTGCTTGACG |
| *Prdx4* | F: GTTTACCCACTTGGCCTGGATT |
|  | R: TCTGCCTCAGGACTCCTTTGT |
| *Prdx5* | F: AGTTCTGTGCTCCGTGCATC |
|  | R: GGCATCTCCCACCTTGATCG |
| *Prdx6* | F: TTGCCTGGAGCAAGGACATC |
|  | R: TAGCGTCCTTCTCGACTGGA |
| *Mrf4* | F: ATCAGCTACATTGAGCGTCTACA |
|  | R: CCTGGAATGATCCGAAACACTTG |
| *Myh3* | F: TCTCTGTCACAGTCAGAGGTGT |
|  | R: GGCTGGTTCTGAGCTTCGAT |
| *Myh4* | F: AGGCTGAGGAGGCTGAGG |
|  | R: TCTCCTGTCACCTCTCAACAGAA |
| *Kif5a1* | F: GCTGTCATCTTGCCAACTGC |
|  | R: CCTCGTGTACAGTTTCGTGC |
| *Kif5b* | F: CCAACTCCGAGCACAAGAGAA |
|  | R: TTGCCTCCACTTCATCTCGC |
| *Dctn1* | F: GGCCTTTGGCTCACAGGTAT |
|  | R: TCTGCCAAGGGGGCAATAAG |
| *Fbxo32* | F: TGAGCGACCTCAGCAGTTAC |
|  | R: GCGCTCCTTCGTACTTCCTT |
| *Trim63* | F: TGCAGAGTGACCAAGGAGAATAG |
|  | R: TTCTCGTCCAGGATGGCGTA |
| *Rhot1* | F: AGGGGGAGGCATGAGACTAC |
|  | R: TTCAGTAGTGCAATCAGGAGGTATT |
| *Trak1* | F: GAGGCTTCTTGAGGAGAAAGAGC |
|  | R: TTCATGGACAGCTCATGTCGG |
| *Mfn1* | F: ATGGCAGAAACGGTATCTCCA |
|  | R: GCCCTCAGTAACAAACTCCAGT |
| *Mfn2* | F: AAGTGGAAAGGCAGGTGTCC |
|  | R: CTGCAGTGAACTGGCAATGG |
| *Opa1* | F: ATACTGGGATCTGCTGTTGG |
|  | R: AAGTCAGGCACAATCCACTT |
| *Fis1* | F: CCGGCTCAAGGAATATGAAA |
|  | R: ACAGCCAGTCCAATGAGTCC |
| *Dnm1l* | F: CTGCCTCAGATCGTCGTAGTG |
|  | R: CGTGGACTAGCTGCAGAATGA |
| *Ppargc1a* | F: ACTATGAATCAAGCCACTACAGAC |
|  | R: TTCATCCCTCTTGAGCCTTTCG |
| *Cytb* | F: AGACAAAGCCACCTTGACCC |
|  | R: GATTGCTAGGGCCGCGATAA |
| *Rplp0* (36B4) | F: AGGATATGGGATTCGGTCTCTTC |
|  | R: TCATCCTGCTTAAGTGAACAAACT |
| 18S | F: CTGCCCTATCAACTTTCGATGGTAG |
|  | R: CCGTTTCTCAGGCTCCCTCTC |

**MOVIES 1-5**

**Movie 1. Nuclear and mitochondrial distribution during *in vitro* myogenesis**

Time-lapse movie showing the differentiation of WT; mt-GFP myoblasts into myotubes over 15 hours. The arrow highlights nuclear centration followed by nuclear spreading.

**Movie 2. Impaired nuclear and mitochondrial spreading in *Prdx5*-knockout myotubes**

Time-lapse movie showing the differentiation of WT; mt-GFP and *Prdx5^-/-^*; mt-GFP myoblasts into myotubes over 22 hours. Myoblasts were transfected with a Lifeact plasmid to visualize the actin cytoskeleton in red. The arrow in WT; mt-GFP indicates the actin-labeled myotube.

**Movie 3. Super-resolution imaging of normal and *Prdx5*-knockout myotubes**

Time-lapse movie captured using Zeiss Elyra 7 with Lattice SIM, showing the differentiation of WT; mt-GFP and *Prdx5^-/-^*; mt-GFP myoblasts into myotubes over 340 minutes. Myoblasts were transfected with a *Map7* plasmid to visualize microtubules in red. Hoechst was used to stain nuclei in blue. Nuclear and mitochondrial spreading are impaired in *Prdx5^-/-^*; mt-GFP myotubes.

**Movie 4. Decreased ATP production and abnormal mitochondrial morphology in *Prdx5*-deficient myotubes**

Short-term continuous live imaging captured using a confocal microscope in Airyscan mode, showing developing WT; mt-GFP, *Prdx5^-/-^*; mt-GFP, and *Prdx3^-/-^*; *Prdx5^-/-^*; mt-GFP myotubes at 24 hours of differentiation. Myotubes were stained with ATP-Red dye to visualize mitochondrial ATP in red. Hoechst was used to stain nuclei in blue.

**Movie 5. Super-resolution imaging of mitochondrial transport in normal and *Prdx5*- or *Trak1*-deficient myotubes**

Short-term continuous live imaging captured using Zeiss Lattice SIM 5, showing 48-hour WT; mt-GFP myotubes transfected with siCtrl, si*Prdx5*, or si*Trak1*. Myotubes were also transfected with a *Map7* plasmid to visualize microtubules in red. Hoechst was used to stain nuclei in blue. Mitochondrial transport along the microtubules is impaired in myotubes deficient in *Prdx5* or *Trak1*.
